# Increased transvascular retention of atherogenic lipoproteins in type 2 diabetes relates to their enhanced proteoglycan binding

**DOI:** 10.1172/jci.insight.177849

**Published:** 2026-04-07

**Authors:** Pär Björklund, Jennifer Härdfeldt, Lauri Äikäs, Sara Straniero, Minna Holopainen, Katariina Öörni, Mats Rudling, Bo Angelin

**Affiliations:** 1 Cardio Metabolic Unit, Department of Medicine Huddinge, Karolinska Institutet, Stockholm, Sweden.; 2 Clinical Department of Endocrinology, Karolinska University Hospital Huddinge, Stockholm, Sweden.; 3 Atherosclerosis Research Laboratory, Wihuri Research Institute, Helsinki, Finland.; 4 Molecular and Integrative Biosciences Research Program, Faculty of Biological and Environmental Sciences, University of Helsinki, Helsinki, Finland.; 5 Helsinki University Lipidomics Unit (HiLIPID), Helsinki Institute of Life Science (HiLIFE) and Biocenter Finland, Helsinki, Finland.

**Keywords:** Clinical Research, Metabolism, Vascular biology, Atherosclerosis, Cholesterol, Lipoproteins

## Abstract

Subendothelial retention of cholesterol-rich apolipoprotein-B–containing lipoproteins drives atherosclerotic arterial disease. In peripheral interstitial fluid from patients with type 2 diabetes (T2D), levels of such particles have been shown to be paradoxically reduced relative to those in serum, presumably reflecting their increased retention within the arterial wall. To identify possible mechanisms involved in lipoprotein retention in T2D, we obtained serum and skin blister fluid from such patients and matched controls, together with skin biopsies in a subset of individuals. In T2D, smaller LDL and VLDL remnant particles were more prominent in serum but not in interstitial fluid, reflecting their enhanced vascular entrapment. The interstitial-fluid-to-serum ratio of apolipoprotein-B was 58% lower in T2D than in controls (0.14 versus 0.33), concomitant with increased susceptibility for LDL binding to proteoglycans. The most marked differences were seen in patients with clinically evident cardiovascular disease. The degree of transvascular retention was positively related to the propensity of isolated serum LDL to bind aortic proteoglycans, both in T2D and in controls. Skin unesterified cholesterol levels were higher in patients with T2D relative to healthy controls. With aging, both proteoglycan binding and apparent vascular retention of LDL increased in controls but not in T2D, indicating that these mechanisms may also be relevant for atherogenesis in nondiabetic individuals.

## Abstract 2

In type 2 diabetes, enhanced susceptibility of serum LDL particles to bind arterial proteoglycans is linked to their increased transvascular entrapment and ultimately atherosclerosis.

## Graphical abstract

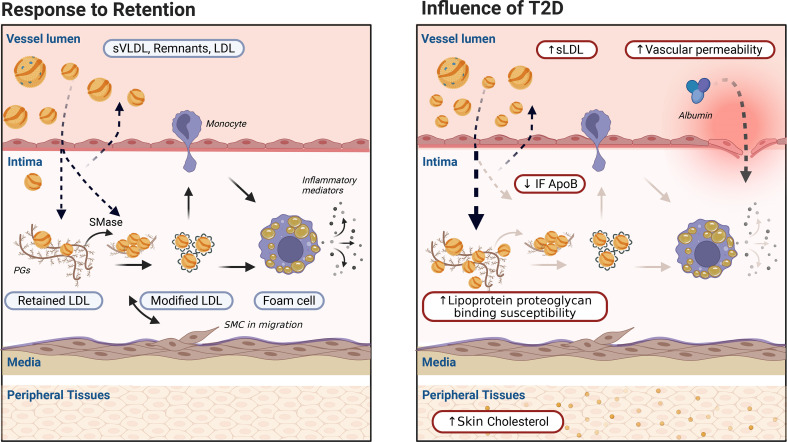

## Introduction

**Figure 1:**
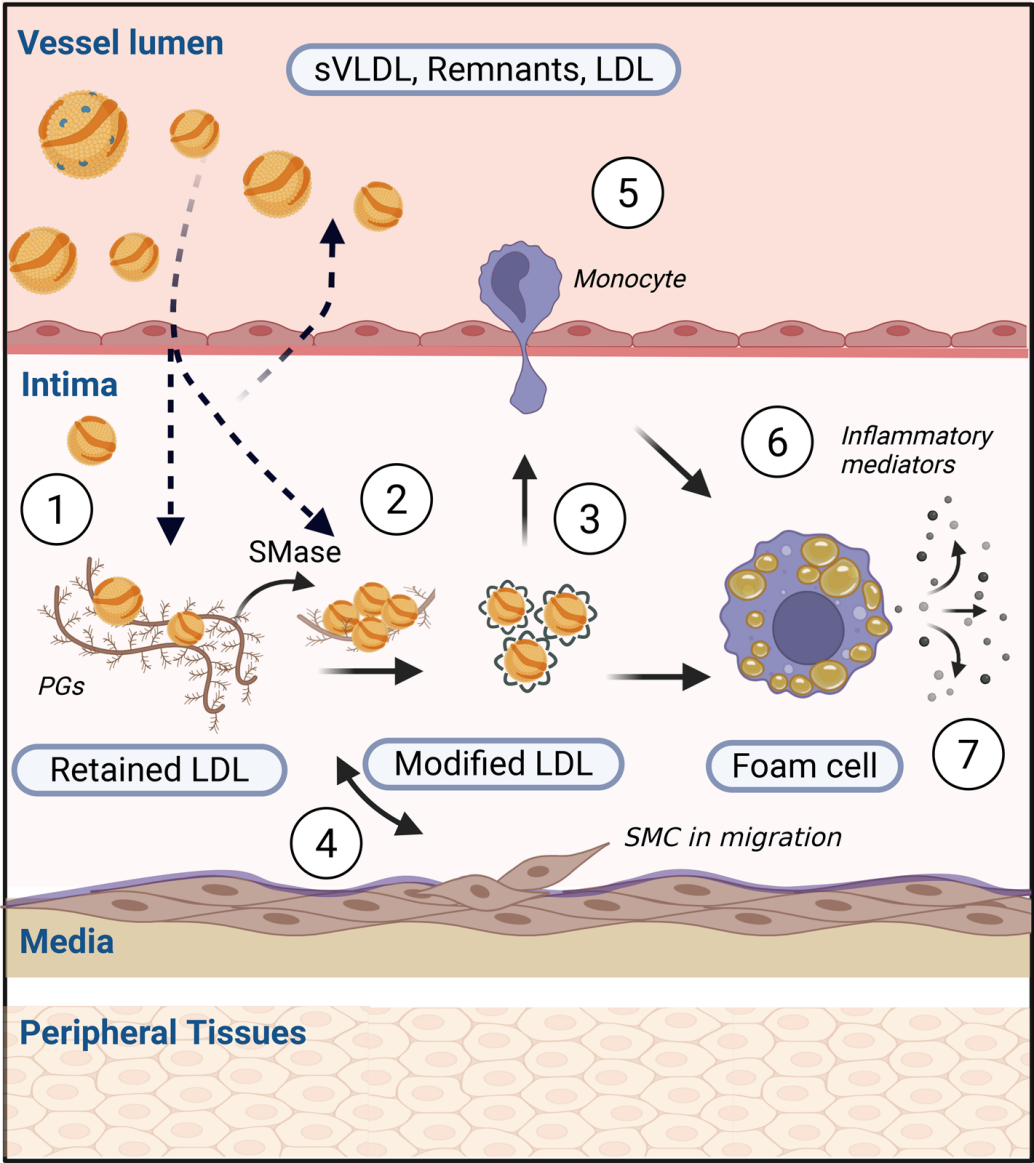
Illustration of the response to retention model of atherogenesis. Initial stages are believed to result from interactions between (label 1) circulating lipoproteins and vascular proteoglycans and (label 2) arterial factors such as secretory sphingomyelinase (SMase) that facilitate lipoprotein aggregation. In label 3, retention of LDL within the subendothelial space leads to increased susceptibility for lipoprotein modification, which triggers (label 4) an inflammatory response involving alterations in smooth muscle cell (SMC) phenotype and function and (label 5) infiltration of immune cells. In label 6, LDL that has been aggregated or modified is avidly phagocytized by macrophages leading to foam cell formation (label 7), which stimulates the release of inflammatory and bridging mediators, further triggering the atherosclerotic cascade (2, 3). Figure created with BioRender.com.

Atherosclerosis is a life-long process of cholesterol accumulation in the subintimal region of arteries, triggering maladaptive inflammatory responses and leading to cardiovascular complications, including myocardial infarction and stroke (1). An important initial step in its pathogenesis is the retention of apolipoprotein-B–containing (apoB-containing) lipoproteins, such as LDL, in the vascular interstitial space, resulting in cholesterol deposition (2–4) (Figure 1). Exploration of the levels and properties of lipoproteins in this compartment of interstitial fluid (IF), which harbors structural and immune cells as well as matrix proteins (5), should be important for the understanding of atherogenesis (6). While direct access to IF in the vascular wall is not possible in humans, studies of lipoproteins in peripheral IF obtained from skin blisters or lymph have shown that their concentrations are approximately 10%–25% of those in serum and that the relative number of particles (HDL>LDL>VLDL) is inversely related to particle size (6–9).

The causal role of LDL in atherogenesis is well established through experimental, genetic, epidemiological, and interventional studies (10, 11). However, in patients with type 2 diabetes (T2D), the risk of atherosclerotic cardiovascular disease is elevated 2- to 4-fold even after adjusting for LDL-cholesterol (12, 13). Although LDL-lowering therapy reduces cardiovascular disease in T2D, a considerable residual risk remains. This is partly due to late initiation of therapies (14) but also indicates that additional proatherogenic processes are present in this disease (13). An increased entry of atherogenic lipoproteins into the subintimal space owing to endothelial dysfunction has been proposed as one such mechanism (15, 16). If present, and of major relevance, such a situation should hypothetically result in increased IF levels of LDL-cholesterol and apoB compared with serum in T2D. However, when this prediction was tested by direct measurements of lipoprotein concentrations in peripheral IF from patients with T2D, their IF/serum (IF:S) ratios were instead markedly lower compared with controls (17). While lipoprotein levels in arterial and peripheral IF are probably not identical (5), it is reasonable to interpret this apparent “net transvascular loss” of atherogenic lipoproteins in T2D as an indirect assessment of enhanced retention within the arterial wall. Such a phenomenon may be the result of altered properties of serum lipoproteins, vascular tissue, and/or the inflammatory response possibly related to this disease (13) (Figure 1).

With the aims to confirm these somewhat paradoxical findings in an additional cohort of patients with T2D, and to further explore possible mechanisms important for the arterial wall retention of atherogenic lipoproteins, we have now addressed several specific questions: (a) Can similar results be obtained also in less severely afflicted individuals with T2D? (b) Can we identify properties of circulating apoB-containing lipoproteins, not only related to their size, lipid composition or modification, but also to their propensity to bind to arterial proteoglycans (18) or to self-aggregate (19), which may relate to the decreased IF:S ratio? (c) Are any of these parameters related to age, sex, BMI, or clinically evident atherosclerosis? (d) Is enhanced retention of atherogenic lipoproteins in T2D reflected by evidence of cholesterol enrichment in peripheral tissues, such as the skin?

## Results

To extend our initial cohort and include more patients representative of patients with T2D treated in primary care, we recruited an additional 40 patients and 39 controls resulting in an extended cohort with a total of 74 patients and 74 controls, respectively (Table 1). Compared with the initial cohort, the newly recruited patients were older and had better metabolic control and shorter disease duration (Supplemental Table 1; supplemental material available online with this article; https://doi.org/10.1172/jci.insight.177849DS1). We were able to confirm that IF:S ratios for atherogenic lipoproteins were reduced also in the less severely afflicted patients (Supplemental Table 2). Furthermore, the high reproducibility of our fast protein liquid chromatography (FPLC) method for serum lipoprotein lipid analyses over time (9) could be confirmed for the initial cohort, in which samples were reanalyzed together with samples from the newly recruited individuals (Supplemental Methods). In the following, all data presented are from the simultaneous determinations in the extended cohort.

As mentioned, vascular permeability is increased in T2D (15, 20, 21), which was confirmed by measuring albumin levels in serum and IF (Table 2). The albumin IF:S ratio was 11% higher in T2D (0.35 ± 0.08 in controls versus 0.39 ± 0.08 versus, *P* < 0.01), indicating an increased endothelial leakage. In controls, but not in T2D, this ratio correlated positively with age and blood pressure (Supplemental Figure 2 and Supplemental Table 3).

**Figure 2:**
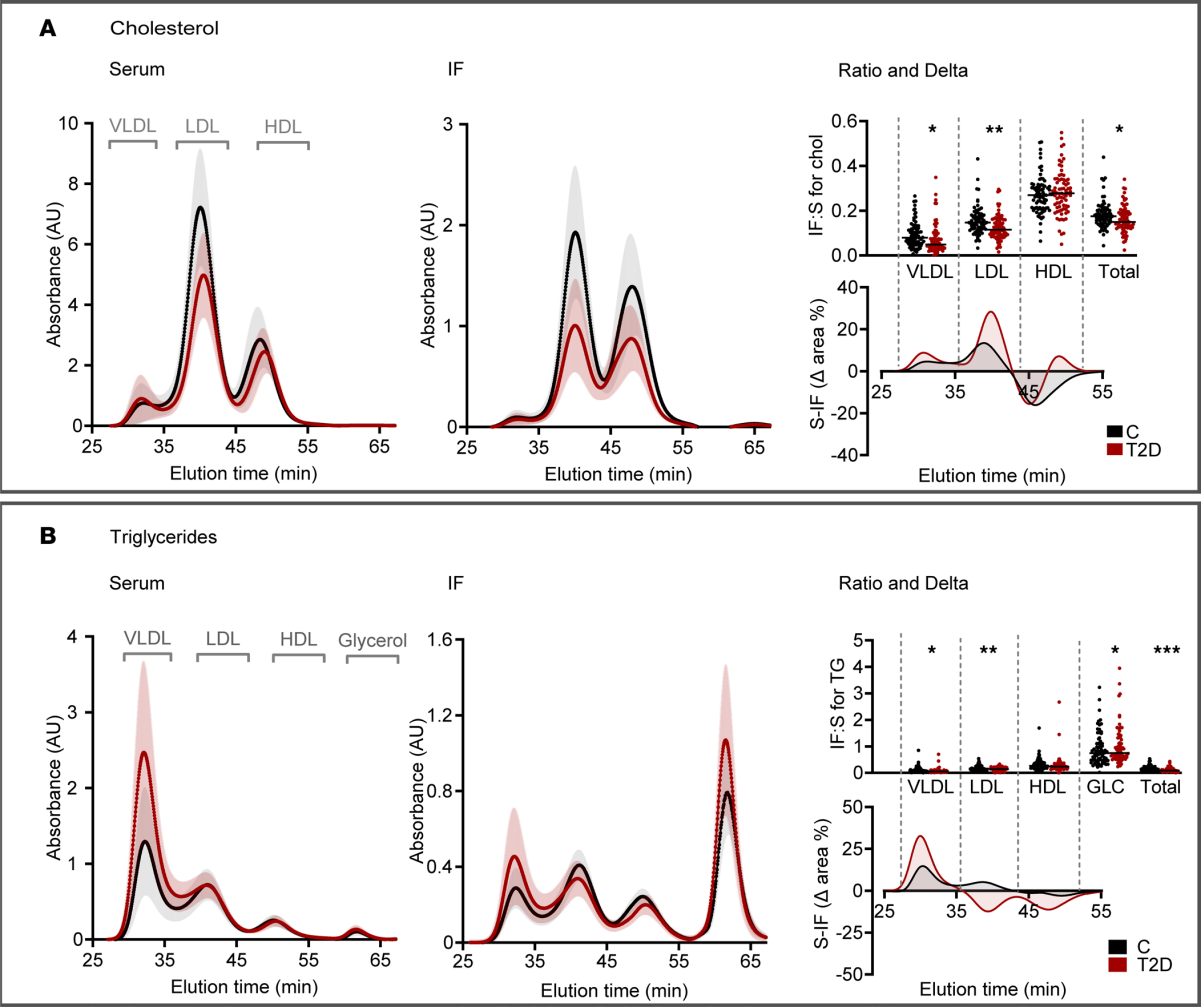
Cholesterol and triglyceride distribution profiles by FPLC. (**A**) Cholesterol (cholesteryl esters + unesterified cholesterol). Lipoprotein profiles for serum and IF, IF/serum ratio (IF:S), and differences between lipoprotein profiles for serum and IF (Δ area%). (**B**) Triglycerides. Lipoprotein profiles for serum and IF, IF/serum ratio (IF:S), and differences between lipoprotein profiles for serum and IF (Δ area%). Data are from 74 patients with T2D and 74 controls and presented as mean ±SEM (shaded areas) and scatter dot plots + mean. Lipoprotein lipids were analyzed by FPLC, and concentrations calculated after integration of the individual chromatograms and adjusted to total cholesterol and triglycerides. IF/serum ratios (IF:S) were calculated by dividing the concentrations of lipoprotein lipids in IF by the corresponding concentrations in serum. The differences between lipoprotein profiles in serum and IF, or serum minus IF (S – IF), were calculated by subtracting area% in IF from area% in serum. Because both serum and IF profiles are normalized to 100%, the S – IF curves reflect relative redistribution across lipoprotein size fractions rather than absolute particle loss; positive values indicate relative depletion from IF and negative values relative enrichment in IF (not analyzed in our previous report; ref. 17). *P < 0.05, ** *P* < 0.01, ***P < 0.001 versus controls (Student’s *t* test). GLC; glycerol.

Continuous size-range analyses utilizing FPLC revealed the typical atherogenic serum lipoprotein pattern seen in T2D (13, 22), including elevated total triglycerides, accumulation of large triglyceride-rich VLDL/remnants, and low HDL-cholesterol (Figure 2 and Supplemental Table 2). Patients with T2D exhibited significantly prolonged elution times for both serum LDL cholesterol (39.3 ± 0.2 min in controls versus 39.8 ± 0.3 min; *P* < 0.001) and HDL cholesterol (49.8 ± 0.6 min in controls versus 50.3 ± 0.6 min; *P* < 0.001), indicating smaller average LDL and HDL particle sizes in patients with T2D. In IF, elution times for cholesterol in apoB-containing lipoproteins (VLDL: 30.2 ± 0.07 min in controls versus 30.1 ± 0.04 min in T2D; LDL: 39.34 ± 0.03 min versus 39.46 ± 0.03 min) did not differ significantly between groups.

Consistent with calibration against published lipoprotein size references (Supplemental Methods), these retention times corresponded to serum LDL particle sizes of 21.0 ± 0.7 nm in controls and 19.7 ± 0.7 nm in T2D, whereas IF LDL particles were of similar size in samples from controls (20.88 ± 0.08 nm) and T2D (20.47 ± 0.09 nm). Serum HDL sizes were estimated at 8.9 ± 0.4 nm in controls and 8.3 ± 0.5 nm in T2D.

The mean IF:S ratio for LDL cholesterol was 20% lower in T2D (0.15 ± 0.06 in controls versus 0.12 ± 0.01, *P* < 0.01). A similar pattern of IF:S ratios was seen for VLDL/remnant particles, whereas there was no difference for HDL-cholesterol (Figure 2A and Table 2). The serum – IF (S – IF) (Δ area%) plots (Figure 2A) confirmed greater proportionate depletions of small LDL and VLDL/remnants from IF in T2D (positive values indicating S% > IF%). In T2D, HDL in IF were instead skewed toward larger particles, suggesting impaired efflux capacity given the higher efflux capacity of small HDL (23). This finding is consistent with our detailed report on the initial cohort (24).

For triglycerides, elution times for lipoproteins did not differ between controls and T2D, and the estimated particle diameters were similarly unchanged (Figure 2B). However, the LDL triglyceride/cholesterol ratio was higher in both serum and IF in T2D than in controls, in agreement with previous reports (25, 26).

**Figure 3:**
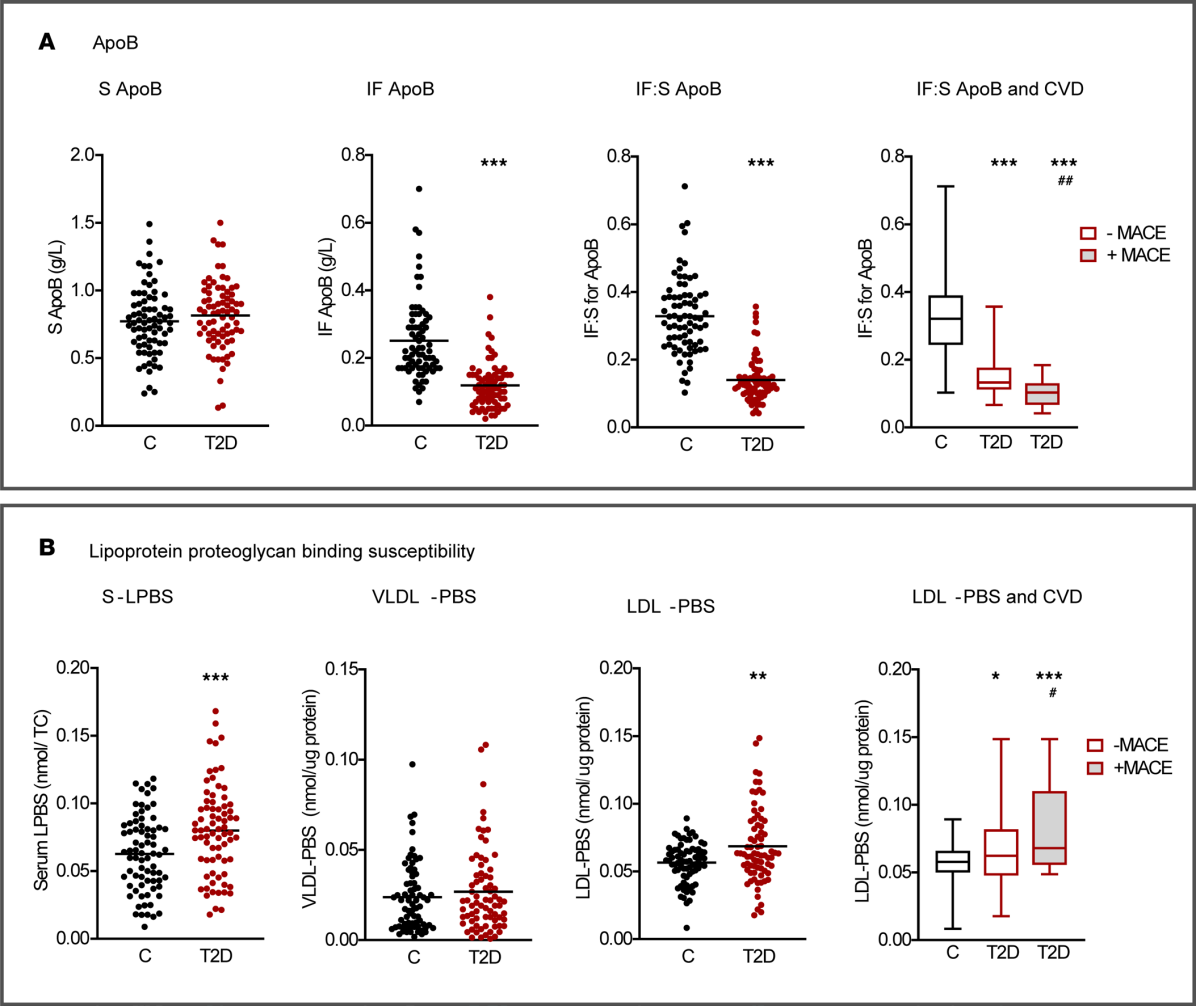
ApoB and lipoprotein proteoglycan binding susceptibility. (**A**) ApoB. Serum, IF, IF:S, and IF:S stratified for MACE. (**B**) Lipoprotein proteoglycan binding susceptibility. Whole serum binding adjusted for serum total cholesterol, isolated serum VLDL and serum LDL binding per µg of protein, and LDL binding stratified for MACE. Data are from 74 patients with T2D and 74 controls and presented as mean (scatter dot plots), mean ±SD, or median (violin plot). (**A**) Controls versus T2D, Student’s *t* test or Mann-Whitney *U* test. (**A** and **B**) C, C+MACE, T2D-MACE, T2D+MACE, 1-way ANOVA with Benjamini and Hochberg FDR correction. *P < 0.05, **P < 0.01, ***P < 0.001 versus controls; ^#^*P* < 0.05, ^##^*P* < 0.01 versus -MACE. CVD, cardiovascular disease; IF, interstitial fluid; IF:S, interstitial fluid-to-serum ratio; LDL-PBS, LDL proteoglycan binding susceptibility; MACE, major cardiovascular event; S-LPBS, serum lipoprotein binding susceptibility; VLDL-PBS, VLDL proteoglycan susceptibility

**Figure 4:**
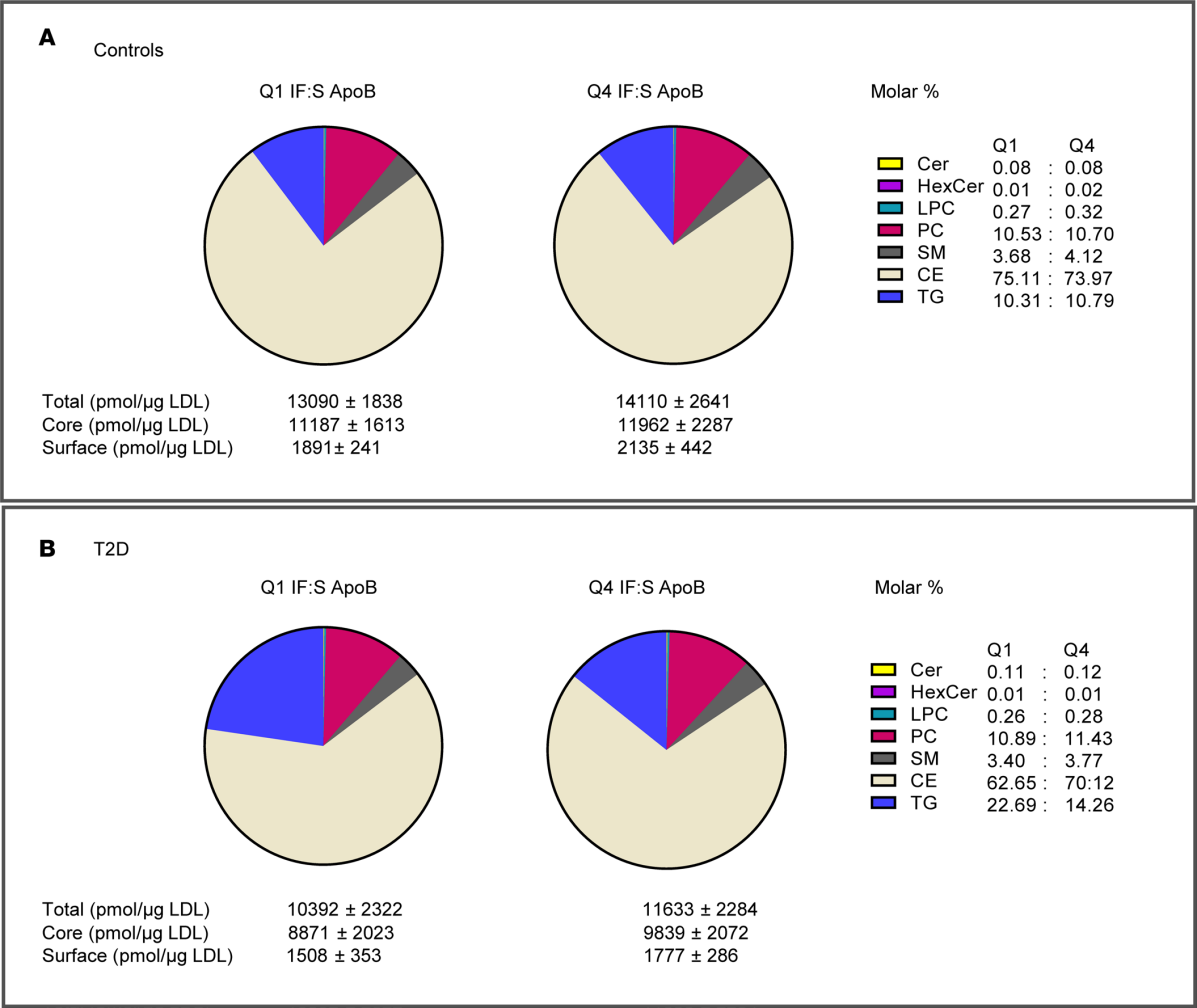
Isolated LDL lipid species. LDL particles were isolated, and their lipid composition was analyzed using mass spectrometry. (**A** and **B**) The total lipid mass and the respective molar percentages (pie graphs) are presented across the lowest and highest 25% of IF:S for apoB for controls (**A**) and T2D (**B**). Lowest 25% ratio [Q1], *n* = 10:8; highest 25% ratio [Q4], *n* = 10:10 (controls:T2D). *P* values of unpaired *t* tests or Mann-Whitney *U* tests were adjusted for multiple comparisons by Benjamini and Hochberg FDR correction. TG, triglycerides; CE, cholesteryl esters; DAG, diacylglycerols; Cer, ceramides; LPC, lysophosphatidylcholines; PC, phosphatidylcholines; PE, phosphatidylethanolamines; PI, phosphatidylinositols; SM, sphingomyelins.

The apoB IF:S ratio was markedly reduced in T2D (0.33 in controls versus 0.14; *P* < 0.001), particularly in patients with clinically evident cardiovascular disease (Figure 3A). To further characterize the LDL particles associated with high apoB depletion in IF, we performed lipidomics analysis on a randomized subset of the extended cohort (Supplemental Methods). In controls, there were no significant differences in cholesteryl ester (CE) or triglyceride composition between individuals with high apoB depletion in IF (lowest quartile [Q1] of apoB IF:S) and those with low apoB depletion in IF (highest quartile [Q4] of apoB IF:S) (Figure 4A and Supplemental Table 4). Serum LDL particles isolated from patients with T2D with a high degree of apoB depletion in IF were significantly depleted in CE (*P* < 0.05) and enriched in triglycerides (*P* < 0.01), compared with serum LDL particles from patients with T2D with a low degree of apoB depletion (Figure 4B and Supplemental Table 4). These findings align with our FPLC data and indicate that smaller LDL particles are preferentially depleted in IF in patients with T2D. Ceramides were significantly enriched in LDL particles from T2D participants compared with the control group with the highest ratio. Other lipid species, and the relative levels of core and surface lipids, did not differ significantly between the low and high binding groups, neither in controls nor in T2D (Figure 4 and Supplemental Table 4).

To explore possible mechanisms involved in the retention of atherogenic serum lipoproteins in T2D (Figure 1), we hypothesized that this was the consequence of a higher susceptibility of such particles to bind to human aortic proteoglycans (3, 18). In accordance with the decreased IF:S ratios for apoB and LDL-cholesterol, the ex vivo binding of serum and isolated LDL from patients with T2D to proteoglycans was increased by 28%, and 20%, respectively, while no difference was seen for VLDL (Figure 3B). The highest binding to proteoglycans was seen in LDL from patients with clinically evident cardiovascular disease, as also observed in early studies by Camejo et al. (27).

**Figure 5:**
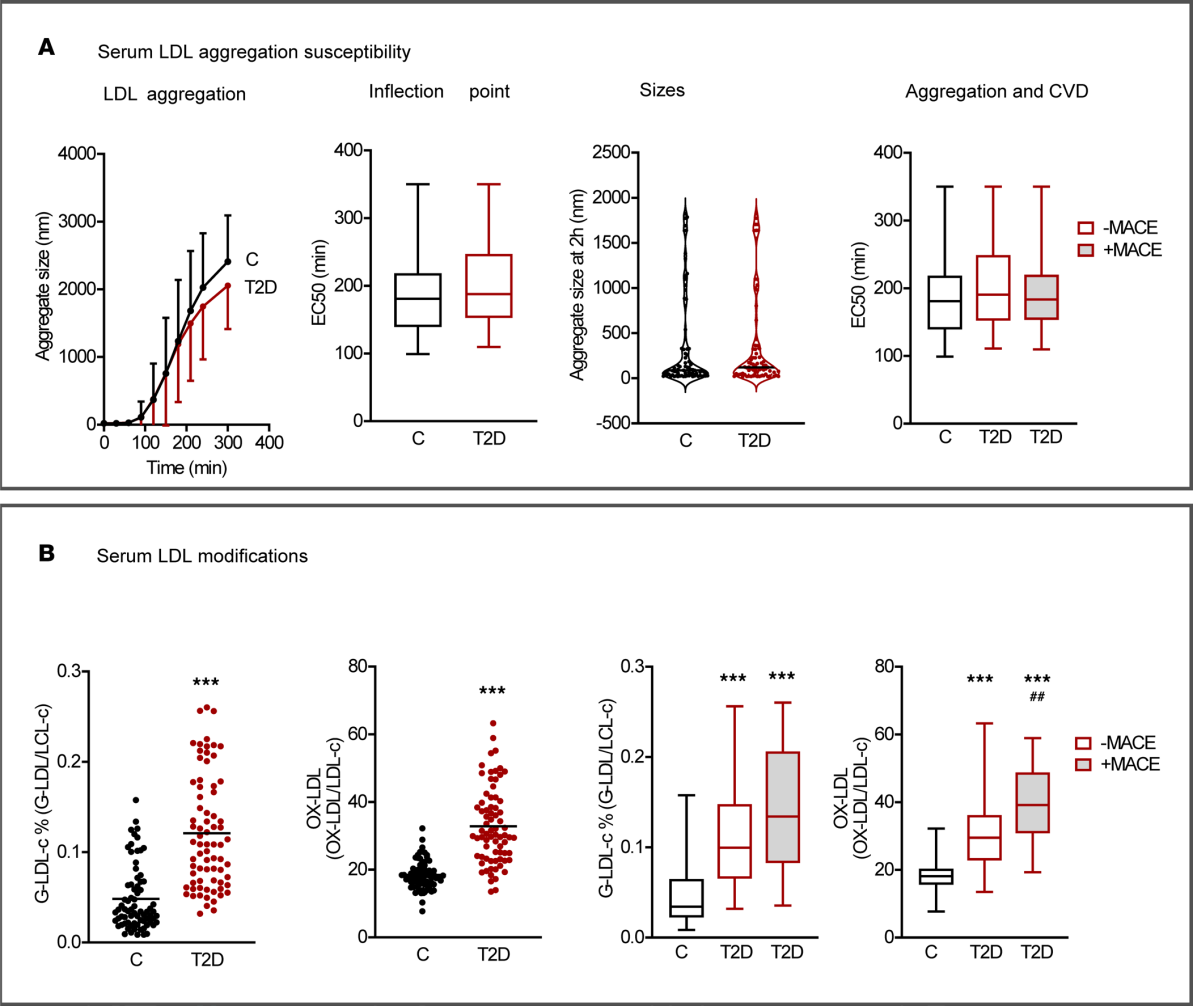
Serum LDL aggregation susceptibility and LDL modifications. (**A**) Serum LDL aggregation susceptibility. LDL aggregation curves: aggregate size (nm) versus time (min) after induction of aggregation with human recombinant sphingomyelinase. Inflection point (EC_50_) of the aggregation curves: the faster the particles aggregate, the shorter the time required to reach the inflection point. Aggregate sizes at 2 h calculated from aggregation curves. EC_50_ of aggregation curves stratified for MACE. (**B**) Serum LDL modifications. %Glycated serum LDL adjusted for LDL cholesterol, serum OX-LDL adjusted for serum LDL cholesterol, %Glycated LDL cholesterol stratified for MACE, and serum OX-LDL adjusted for LDL cholesterol stratified for MACE. Data are from 74 patients with T2D and 74 controls and presented as mean ± SD (LDL aggregation curves), median (violin plot), or median, upper and lower quartiles, and range (box-and-whisker plots). Controls versus T2D, Student’s *t* test or Mann-Whitney *U* test. C, C+MACE, T2D-MACE, T2D+MACE, 1-way ANOVA with Benjamini and Hochberg FDR correction. ***P < 0.001 versus controls; ^##^*P* < 0.01 versus –MACE. MACE, major cardiovascular event; OX-LDL, oxidized LDL.

Exposure of LDL particles to lipoprotein modifying enzymes, such as sphingomyelinase, promotes their aggregation and fusion (Figure 1). We therefore tested if serum LDL from patients with T2D were more susceptible to self-aggregation following exposure to human recombinant secretory acid sphingomyelinase (19, 28). No such differences, however, were observed for any comparison of aggregation susceptibility that we made between serum LDL from controls versus patients with T2D (Figure 5A and Supplemental Figure 3C).

To further characterize possible metabolic properties of the atherogenic LDL particles from T2D serum involved in proteoglycan binding and apparent vascular retention, we assessed the extent of glycation or oxidation. As anticipated, patients with T2D displayed notable increases in these modifications compared with controls (Figure 5B).

**Figure 6:**
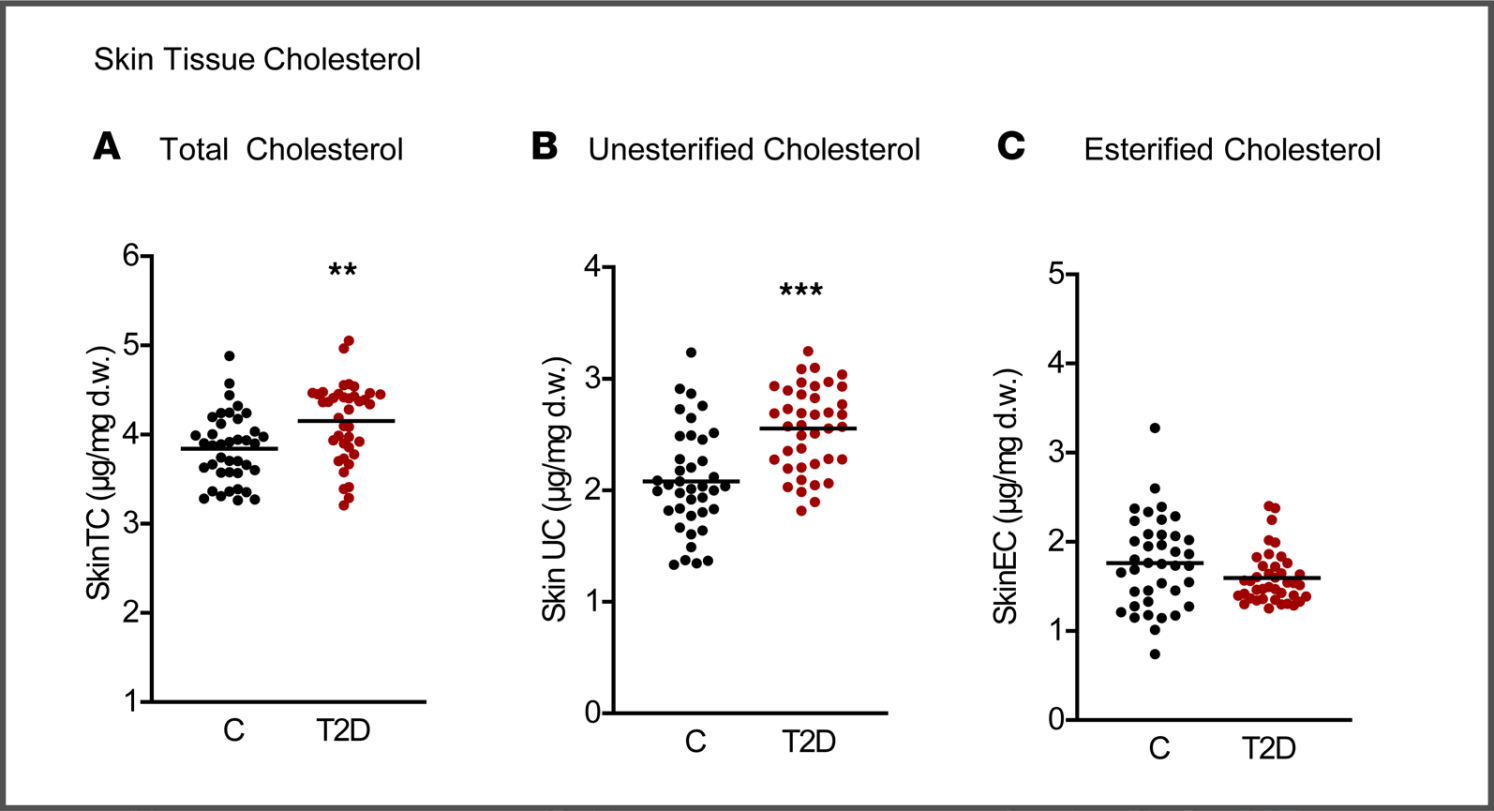
Skin tissue cholesterol. (**A**) Total cholesterol. (**B**) Unesterified cholesterol. (**C**) Esterified cholesterol. Cholesterol was assayed by GC/MS after addition of deuterium-labeled cholesterol as internal standard. Unesterified cholesterol was determined by the same method, but the hydrolysis step was omitted. The concentration of esterified cholesterol was calculated as the difference between the total and the unesterified cholesterol. Skin tissue cholesterol was adjusted for dry weight of biopsies (μg cholesterol/mg dry weight) and data are presented as mean with scatter dot plots. **P < 0.01, ***P < 0.001. Controls versus T2D, Student’s *t* test.

Because our results suggest an enhanced vascular entrapment of atherogenic lipoproteins in T2D, we hypothesized that these patients would also have an increased cholesterol deposition in peripheral tissues. This was tested through analyses of skin biopsies obtained from a subset of the extended cohort. In this limited series, levels of total cholesterol were 8% higher in T2D (3.84 ± 0.38 in controls versus 4.15 ± 0.43 μg/mg dry weight [d.w.]), reflecting a 23% (2.08 ± 0.46 in controls versus 2.56 ± 0.37 μg/mg d.w.) increase in unesterified cholesterol in T2D (Figure 6).

**Figure 7:**
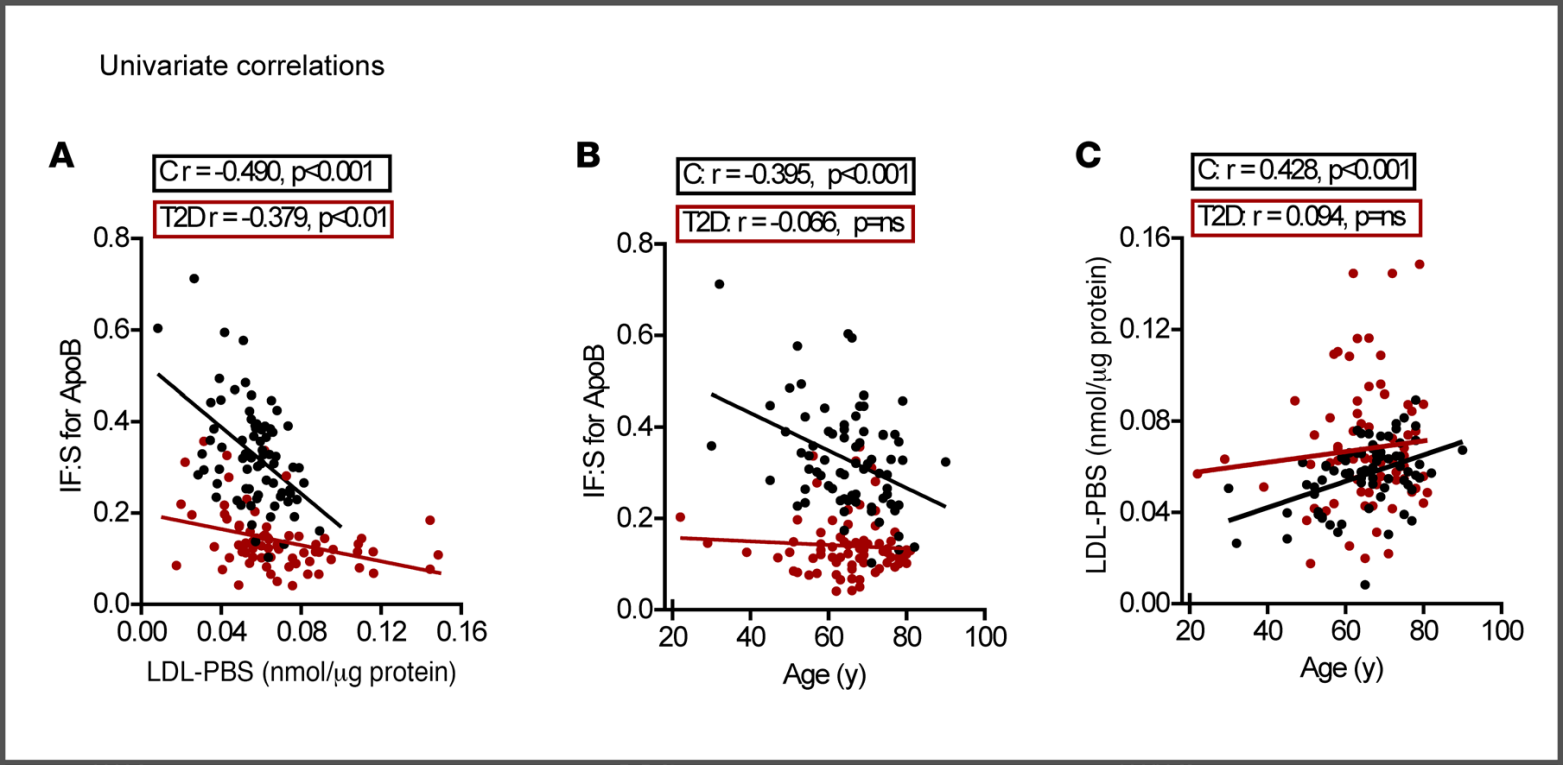
Graphic representation of univariate correlations. (**A**) Relationship between proteoglycan binding to LDL and IF:S for apoB. (**B**) Relationship between age and IF:S for apoB. (**C**) Correlation between age and proteoglycan binding to LDL. Data are from 74 patients with T2D and 74 controls. Pearson correlation coefficients were obtained from univariate regression analyses; *P* < 0.05 indicates statistical significance. IF:S, interstitial fluid-to-serum ratio; LDL-PBS, LDL proteoglycan binding susceptibility.

While the differences in IF:S ratios of atherogenic lipoproteins between controls and patients with T2D were clear, it was also obvious that there were substantial interindividual variations within both groups. To test the hypothesis that the propensity of serum LDL to bind proteoglycans modulates their degree of depletion when traversing from serum to IF, we predicted that this property would be negatively related to the IF:S ratio of apoB. This was clearly the case in T2D (Figure 7A and Supplemental Table 3), and this relationship was even more obvious in the controls. Similar relationships of LDL proteoglycan binding, although less pronounced, were also observed for IF:S ratios of LDL-cholesterol (Supplemental Table 3). While we did not observe any sex-related differences (Supplemental Figure 3), age correlated negatively to IF:S ratio of apoB and positively to LDL proteoglycan binding in controls, but not in patients with T2D (Figure 7, B and C*)*. LDL from patients with macrovascular disease exhibited the lowest IF:S ratio of apoB and the highest susceptibility to bind proteoglycans, whereas no differences in either ratio or binding were observed between those with and without microvascular complications (Supplemental Figure 3).

To further explore which parameters associate with the IF:S ratios of apoB, we conducted multiple regression analyses. Using the full extended cohort, we identified T2D, proteoglycan binding (LDL-PBS), and age to best account for the variation in the IF:S ratio of apoB. All these parameters were independent of lipoprotein levels. T2D had the largest statistical effect, followed by serum LDL-PBS and age. HbA1c, time since T2D diagnosis, sex, systolic blood pressure, cystatin C, hsCRP, statin treatment, and serum non-HDL cholesterol were excluded from the final model because they were not statistically significant in the univariate analysis (*P* > 0.05). BMI, glycated LDL, and OX-LDL/LDLc were associated with the apoB ratio in univariate analyses but did not remain independent predictors in multivariable models, nor did they materially alter the effect estimate for T2D. The final model showed a strong fit, accounting for 60% of the variation in the IF:S ratio of apoB.

## Discussion

**Figure 8:**
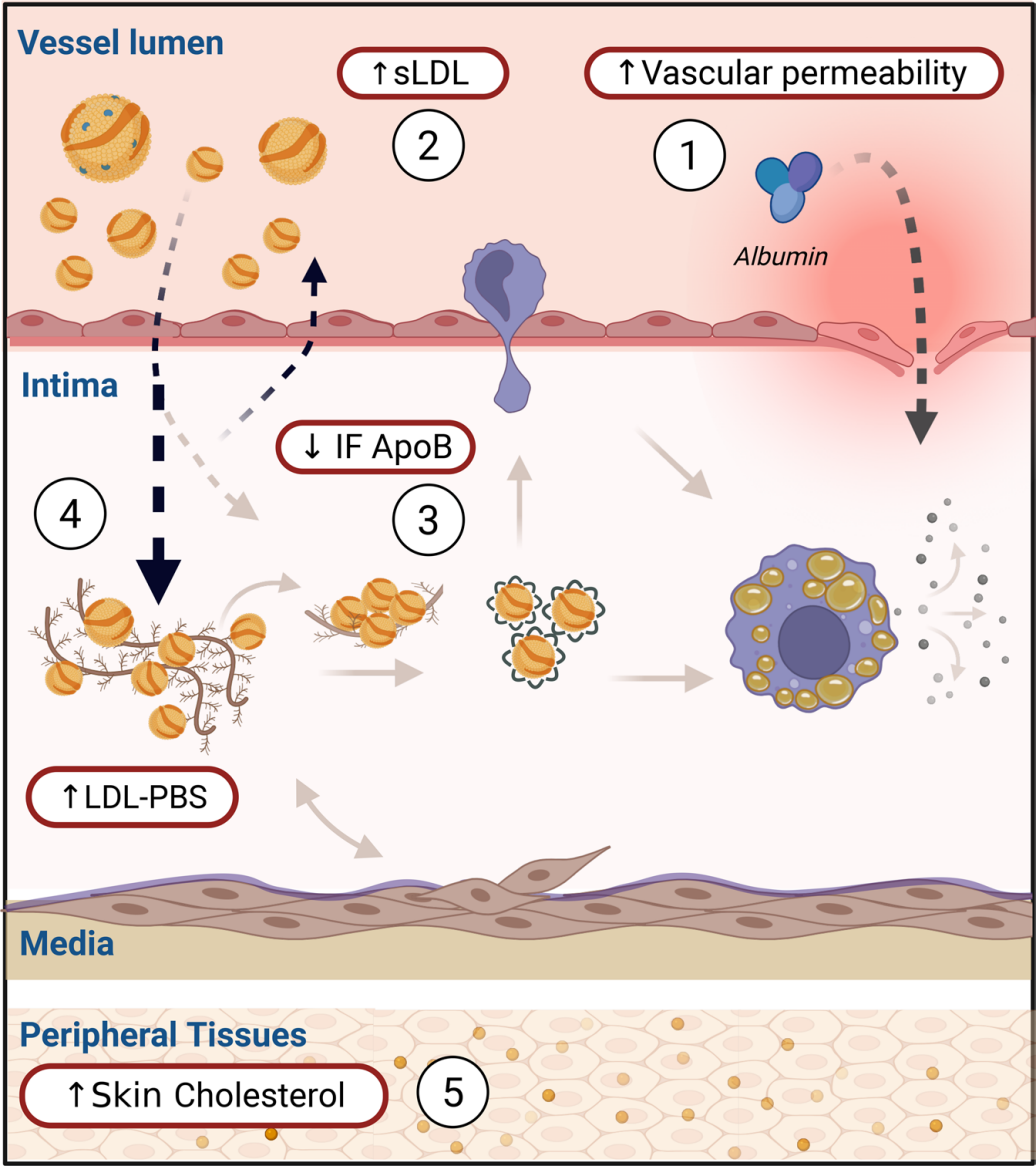
Influence of T2D on response to retention. In label 1, functional and structural changes in the endothelial barrier function. In label 2, preponderance of proatherogenic small LDL in serum. In label 3, selective reduction of atherogenic lipoproteins in interstitial fluid (IF) following retention of small LDL and remnant particles due to (label 4) increased proteoglycan binding susceptibility of serum LDL particles. While there were no differences in LDL aggregation in serum, vascular retention of apoB-lipoproteins may still promote local modification and downstream inflammatory effects not captured by our serum-based aggregation assay. Increased deposition of unesterified cholesterol could be demonstrated in skin biopsies (label 5). A similar but less pronounced pattern (labels 1–5) was seen with increased age in the control group. Figure created with BioRender.com.

In this cross-sectional study, we focused on the potential significance of the levels and properties of atherogenic lipoproteins in serum and IF in the context of T2D, associated with increased atherosclerosis and cardiovascular morbidity. Several key conclusions emerge from our findings which merit further discussion (Figure 8).

First, we confirmed in a new cohort that the concentrations of apoB-containing lipoproteins, particularly LDL, are lower in IF from patients with T2D when compared with matched healthy controls, also in patients with a less severe T2D phenotype. This difference was most pronounced for small LDL particles, which were increased in T2D serum but not detected in IF. Small LDL particles are recognized as a hallmark of diabetic dyslipidemia and have been implicated in atherogenesis, based on their presumed longer half-life, increased transport into the subendothelial space, and susceptibility to modifications (18, 22, 29). To estimate lipoprotein retention in the arterial wall, we relied on the assumption that measurements in skin blister fluid reflect IF composition. While obviously an indirect assessment, calculation of the S – IF differences for different lipoproteins demonstrated that small LDL and remnant particles represent the major fractions of serum lipoproteins that are depleted when traversing from the bloodstream into the IF, also in healthy individuals (Figure 2). The reduction of small LDL and VLDL remnant particles in IF from patients with T2D may intuitively be seen as paradoxical, since smaller lipoproteins should pass more readily from the bloodstream into IF. However, it is also important to acknowledge that our explorative approach most likely underestimates the true extent of lipoprotein retention in T2D, since simultaneous endothelial “leakiness” for serum proteins (20) — as demonstrated here for albumin — probably also involves small LDL and other lipoproteins (15, 16).

Second, whole serum and isolated LDL from patients with T2D, particularly those with clinically evident atherosclerotic cardiovascular disease, displayed increased binding to arterial proteoglycans ex vivo. This property was inversely correlated with the IF:S ratio of apoB in both patients with T2D and controls, apparently explaining the paradoxical finding of reduced IF levels of apoB and providing support for its relevance in early vascular retention (3, 27, 30). Our present results agree with previous studies reporting increased LDL proteoglycan binding in individuals with smaller-sized LDL, including patients with diabetes and the metabolic syndrome (18, 30, 31).

Previous studies have shown that additional qualitative modifications of LDL such as glycation may further enhance proteoglycan binding (32–34), while oxidation has been reported to reduce the interaction of LDL with proteoglycans (35). In our study, the percentage of glycated LDL showed a significant negative correlation with the IF:S ratio of apoB and a significant positive correlation with LDL-PBS in patients with T2D in univariate correlation analyses. The proportion of oxidized LDL demonstrated a significant negative correlation with IF:S ratio of apoB but was not associated with LDL-PBS (Supplemental Table 3). In the final multiple regression model of the entire extended cohort (*n* = 148), neither glycated LDL nor oxidized LDL provided significant additional predictive power for the IF:S ratio of apoB (Table 3).

Beyond LDL, Lp(a), another highly atherogenic apoB-containing lipoprotein, may also contribute to vascular lipoprotein retention and atherogenesis through its content of biologically active lipids and structural homology with LDL (36). However, in our initial cohort (17) Lp(a) did not differ in serum or IF concentrations, or in IF:S ratios between patients with T2D and controls. Lp(a)-specific mechanisms were not explored further here but warrant future investigations, particularly regarding the marked interindividual variations in Lp(a) phenotype (36).

Additional possible contributors to apoB retention in T2D, also not examined here, include alterations in vascular proteoglycans, dysfunctional HDL, and variations in apoC3 and phospholipase A2 (31, 37–40). Recent work on the response to retention hypothesis highlights that diabetes-associated changes in glycosaminoglycans and other matrix components can enhance LDL binding capacity in the arterial wall (41). To define disease-specific mechanisms of apoB lipoprotein retention, including for oxidatively modified LDL, future studies should therefore compare proteoglycans isolated from atherosclerotic plaques in individuals with and without T2D, as well as from nonatherosclerotic arteries. In addition, other extracellular matrix components, such as collagens and elastin, may modulate LDL retention (42).

As expected, most patients with T2D included were on statin treatment, which may have influenced comparisons with controls. However, in a small, randomized trial (17), lipoprotein IF:S ratios did not change following short-term treatment with atorvastatin, and in the present study, statin treatment was not independently associated with LDL proteoglycan binding (Table 3 and Supplemental Figure 3). Statin treatment has been reported to decrease LDL proteoglycan binding in some studies (43) but not consistently (44). If present, however, such effects would result in underestimation of the degree of apparent lipoprotein retention in T2D with our technique.

Third, in contrast to the influences on apparent lipoprotein retention exerted by proteoglycan binding, no differences were seen between T2D and controls for serum LDL aggregation susceptibility in the presence of secretory acid sphingomyelinase, and this property was not related to sex, age, medication, glycemic control, or diabetic complications. While LDL aggregation susceptibility promotes foam cell formation, activates inflammatory response, and predicts future events in cardiovascular disease (28, 45), our data do not implicate this property of LDL as a primary driver of lipoprotein retention in T2D. In agreement with previous studies (28), aggregation measurements displayed large interindividual differences, and these differences likely affect early and later stages of atherogenesis (Figure 8). Owing to the small volumes of IF, we were not able to analyze aggregation or proteoglycan binding in LDL isolated from this compartment. In addition, we were not able to examine whether proteoglycan-binding influenced the susceptibility of LDL for aggregation, as has been reported (46).

Fourth, in healthy controls, increasing age was significantly correlated with both reduced IF:S ratios of apoB and increased LDL binding to arterial proteoglycans, supporting the concept that the latter may also be relevant for atherogenesis in nondiabetic individuals. The aging process is frequently linked to metabolic perturbations, including the development of obesity, insulin resistance, and reduced endothelial function, each of which has been linked to accelerated atherosclerosis (47). The lack of influence of age on lipoprotein retention or proteoglycan binding in T2D suggests that the processes enhancing LDL-PBS interactions in patients with T2D are already fully active, independent of chronological age. The multiple regression model was unable to effectively capture this key observation owing to high multicollinearity when introducing an age × T2D interaction term. The issue persisted even after attempts to mitigate multicollinearity through standardization and other transformations.

Fifth, based on the finding of increased apparent retention of atherogenic lipoproteins in T2D, we predicted that this may lead to evidence of cholesterol accumulation in peripheral tissues. We tested this prediction in a limited number of individuals and observed higher levels of unesterified cholesterol in skin biopsies from subjects with T2D than in healthy controls, consistent with earlier reports of parallel increases in skin and arterial cholesterol with aging and atherosclerosis (48–50). Elevated skin unesterified cholesterol has also been described in human and experimental inflammatory skin disease, including clinically unaffected areas, where macrophages display an increased propensity to form cholesterol crystals (51, 52). Together with evidence that extracellular apoB-containing lipoproteins in atherosclerotic lesions are enriched in unesterified cholesterol and promote extracellular crystal formation, these observations support a model in which enhanced extralysosomal hydrolysis of aggregated lipoprotein CE by macrophages drives excess free cholesterol (19, 53, 54).

While our work has its strengths by replicable results obtained in humans using established methodology in samples from well-characterized patients and controls, its limitations are related to the difficulties in finding drug naive patients with T2D, and in obtaining IF truly representative of the arterial wall, thus making quantitative analyses difficult. Our work also raises several important questions to be further explored. One pertains to whether a potential inflammatory response triggered by increased cholesterol deposition in tissues can be detected in IF from patients with evidence of greater apoB-lipoprotein retention or increased propensity of apoB-lipoproteins to aggregate (1, 28). Comparing IF lipoproteins from asymptomatic patients with and without atherosclerosis, and from patients with various monogenic diseases influencing serum LDL levels should also be of interest. Characterizing IF lipoproteins in the postprandial state should contribute to the understanding of their possible role in atherogenesis, as should testing the predictive value of IF:S ratios in prospective studies on incident cardiovascular disease.

Although there are well-known species differences regarding lipoprotein metabolism and atherosclerosis, studies of the metabolism of various lipoproteins isolated from serum and IF in suitable animal models (55) may be useful to characterize their interaction with the arterial wall in diabetes. A key limitation is that arterial LDL retention in T2D versus controls was not measured directly; instead, we relied on the IF:S ratio and in vitro proteoglycanbinding assays as indirect proxies. Future studies using direct approaches, such as labeled LDL tracking or advanced imaging and arterial uptake studies in suitable animal or ex vivo human models, will be important to confirm differential LDL retention in T2D.

Finally, it is tempting to speculate that some of the 3-fold interindividual variation in LDL proteoglycan binding observed in both patients with T2D and controls could be the consequence of genetic variation — e.g., polymorphisms in the *APOB* gene sequence related to the proteoglycan binding site of this protein (56, 57).

In conclusion, the significantly lower concentration of apoB-containing lipoproteins in peripheral IF relative to serum in patients with T2D occurs in parallel with an increased susceptibility of their LDL particles to bind to arterial proteoglycans. Together with the preliminary finding of higher tissue cholesterol levels, our data support the concept of enhanced retention of atherogenic lipoproteins as a key feature of the process of accelerated atherosclerosis in T2D. Similar changes were observed with increasing age in control patients, suggesting that such mechanisms may be of relevance also in nondiabetic individuals. Further investigations of IF and serum lipoproteins should have the potential to provide valuable insights into the pathogenesis of human atherosclerosis, and eventually its prevention and treatment.

## Methods

### Sex as a biological variable.

This study recruited both males and females and similar findings are reported for both sexes.

### Study design and population.

In order to obtain samples from a wider age group, and from patients with T2D more similar to the average T2D patient in Sweden compared with the relatively severely afflicted patients included in our previous report (17), we recruited additional individuals from primary care centers, as well as age- and sex-matched healthy controls, from among members of a sports center and an order society for seamen and sea captains, all in Stockholm, Sweden.

Exclusion criteria for the newly recruited individuals were in line with the ones for the initial cohort (Supplemental Figure 1), and collection of data was carried out between January 2018 and December 2019 at the clinical science center at Karolinska University Hospital Huddinge. Patients arrived in the morning after an overnight fast. Height, weight, and bioimpedance measurements were registered. Blood pressure was measured in the sitting position. Phlebotomy was performed, and blood was sent to the central hospital laboratory for routine analysis or used for plasma and serum isolation and immediately frozen in aliquots at −80°C. Further details are given in Supplemental Methods.

In the extended cohort, 90% of patients with T2D were on long-term antidiabetic treatment (Table 1) (metformin *n* = 51; insulin *n* = 33; GLP1-agonist *n* = 6, SGLT2-inhibitor *n* = 2, DPP4-inhibitors *n* = 10; sulfonylurea *n* = 9; glitazone *n* = 9), while 74% of the patients with T2D were on lipid lowering therapy (simvastatin *n* = 26, atorvastatin *n* = 24, rosuvastatin *n* = 5, ezetimibe *n* = 5, fibrate *n* = 1). Altogether, 72% of the patients with T2D had pathological HbA1c (>48 mmol/mol), 27% had experienced a prior major adverse cardiovascular event (MACE; acute myocardial infarction *n* = 16, stroke *n* = 5), and 53% had microvascular complications, including 16% with impaired kidney function.

### Collection of IF and skin biopsies.

Suction blisters were generated in fasting patients ad modum Kiistala (58) as described (17). Three 4 mm punch biopsies were taken under local anesthesia from abdominal skin lateral to the position of the cups from 39 patients with T2D and 39 controls. S.c. fat was separated from the dermis and epidermis under a microscope; the biopsies were then snap frozen. Detailed descriptions of procedures are given in Supplemental Methods.

### Laboratory analyses.

Serum and IF lipoproteins were analyzed by FPLC (9). Analyses of apolipoproteins, albumin, glycated LDL, and oxidized LDL were performed with commercially available kits (Supplemental Methods). Serum lipoproteins for ex vivo assays were isolated by sequential deuterium oxide–based ultracentrifugation at 40,000 rpm (172,000*g* average) at 4°C (59). Solid-phase assays were used to quantitate binding of apoB-containing lipoproteins to human aortic proteoglycans (44, 60). The aggregation susceptibility of isolated LDL was determined in vitro using sphingomyelinase-induced aggregation assay (28, 61). For lipidomics analysis of isolated LDL particles from randomly selected individuals, from the lowest and highest 25% of IF:S ratio for apoB, were quantified using mass spectrometry. Lipid extracts from skin biopsies were assayed for unesterified and total cholesterol using gas chromatography/mass spectrometry. Detailed descriptions and references of laboratory procedures are given in the Supplemental Methods.

### Statistics.

Numerical parameters are presented as mean ± SD except in Figure 2, where mean ± SEM is used (shaded) to illustrate the precision of the group comparisons. Categorical data are presented as numbers and percentages. Data on triglycerides were log_2_ transformed prior to statistical analysis to achieve normality. Missing data on lipoprotein proteoglycan binding and skin cholesterol were regarded as missing at random (MAR) and resulted in exclusion (Supplemental Figure 1). Missing data on HbA1c (1 patient with T2D) and hsCRP (1 control) were regarded as MAR; statistical analyses were performed using pairwise deletion. Unadjusted pairwise comparisons between 2 independent groups were conducted using 2-tailed Student’s *t* test, or 2-way ANOVA for more than 2 groups. Tukey’s Honestly Significant Difference (HSD) test was used for post hoc analysis of lipidomics data following ANOVA. Shapiro-Wilk and visual inspection of quantile-quantile (QQ) plots were used for assessment of normality, and Pearson’s correlation coefficient was used for all correlation analyses.

In the multiple linear regression modeling, independent parameters were selected based on their clinical relevance and statistical significance in univariate analysis. Model fit was evaluated by residual plots, QQ plots, R^2^, adjusted R^2^, Akaike’s Information Criterion (AIC), and Bayesian Information Criterion (BIC) as recommended in standard regression diagnostics (62). Residuals were found to be normally distributed around zero. There was no significant multicollinearity among independent parameters. Results are reported as β coefficients with 95% CIs. Statistical analyses were conducted using established principles for medical research (63).

Two-sided *P* values are reported. *P* < 0.05 was considered significant. We used GraphPad Prism9 for Student’s *t* test and ANOVA, and for making tables and graphics; STATAMP 15.1 was used for multiple regressions and power calculations. Detailed descriptions are given in Supplemental Methods.

### Study approval.

The extended study was approved by the regional ethics review board in Stockholm (registration no. 2017/1942-3, 2018/1428-32) and conducted in accordance with the Declaration of Helsinki. Written informed consent was obtained before inclusion. This study was registered at ClinicalTrials.gov with identifier NCT03386097. Further details are given in Supplemental Methods.

### Data availability.

Data supporting the findings of this study are available in the Supporting Data Values.

## Author contributions

BA and MR conceptualized the study and designed the research questions. PB and JH contributed to the conception and design of the study, acquisition of data, analysis, and interpretation. LÄ and MH contributed to acquisition of data and data curation. BA, MR, SS, and KÖ designed the methodology and supervised the findings of the study. BA, MR, and KÖ acquired funding for the study. PB, JH, and BA wrote the original draft, and all authors contributed important intellectual content to the final manuscript. PB and JH are co–first authors, order decided by coin toss.

## Conflict of interest

BA consults for and receives grants from AstraZeneca, Albireo/Ipsen, and Dicot Pharma. KÖ is an inventor in a patent on measurements of LDL aggregation (no. P4629US01).

## Funding support

Supported by grants from the following organizations:

- The Swedish Heart-Lung Foundation (grant no. 20200736) to BA
- Swedish Research Council (grant no. 521-2013-2804) to BA
- Knut and Alice Wallenberg Foundation (grant no. 2015.0298) to BA
- Stockholm City Council/ALF (grant no. FoUI-975446, 20180339) to BA and MR
- Academy of Finland (grant no. 332564) to KÖ
- Novo Nordisk Fonden (grant no. NNF19OC0057411) to KÖ
- CIMED (grant no. FoUI-963434) to BA
- KI foundations (grant no. 2018-01604, 2020-02414) to BA and JH
- The Finnish Foundation for Cardiovascular Research to KÖ
- Aarne Koskelo Foundation to KÖ
- Sigrid Jusélius Foundation KÖ
- The KI/AZ Integrated Cardio Metabolic Center to BA, MR, PB, JH, and SS

## Supplementary Material

Supplemental data

Supporting data values

## Figures and Tables

**Table 1:**
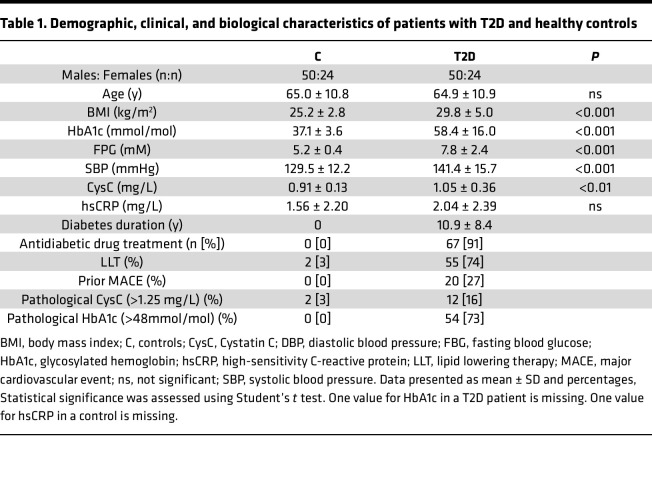
Demographic, clinical, and biological characteristics of patients with T2D and healthy controls

**Table 2:**
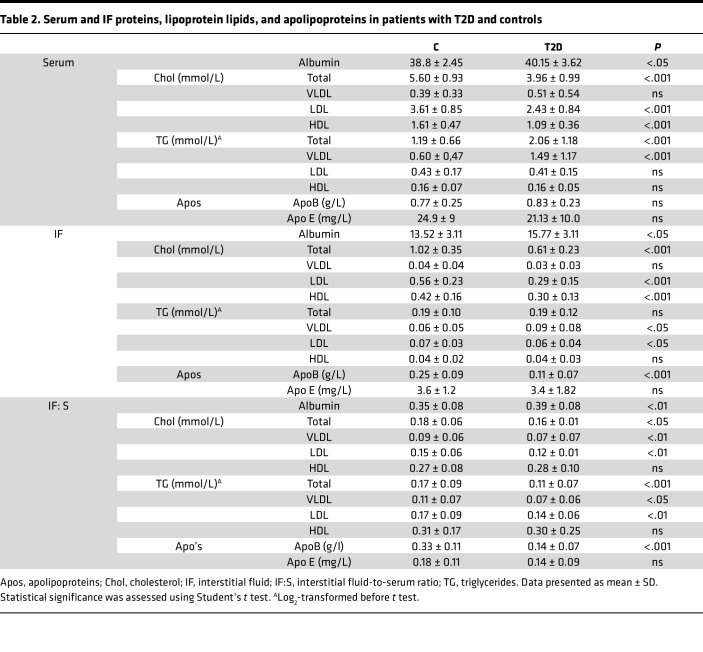
Serum and IF proteins, lipoprotein lipids, and apolipoproteins in patients with T2D and controls

**Table 3:**
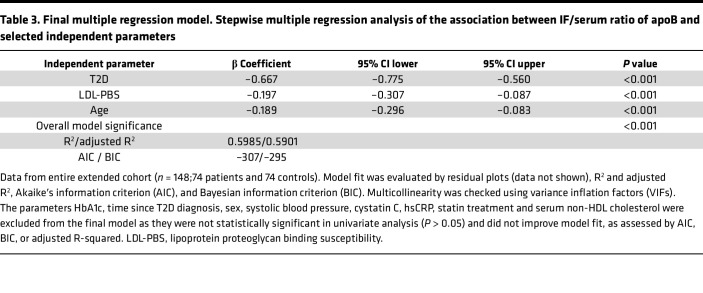
Final multiple regression model. Stepwise multiple regression analysis of the association between IF/serum ratio of apoB and selected independent parameters

## Version 1 04/07/2026

In-Press Preview

## Version 2 05/22/2026

Electronic publication
