# Supplementary material for: Increased transvascular retention of atherogenic lipoproteins in type 2 diabetes relates to their enhanced proteoglycan binding
Source: JCI Insight. 2026 Apr 7;11(10):e177849. doi: 10.1172/jci.insight.177849 (PMC13232734; doi:10.1172/jci.insight.177849)
Supplement: Supplemental data [file jciinsight-11-177849-s210.pdf]

SUPPLEMENTARY MATERIAL

Increased transvascular retention of atherogenic lipoproteins in type 2 diabetes relates to their enhanced proteoglycan-binding susceptibility

Pär Björklund[1,2*], Jennifer Härdfeldt [1,2*], Lauri Äikäs [3,4], Sara Straniero [1,2], Minna Holopainen [4,5]

Katariina Öörni [3,4], Mats Rudling [1,2], Bo Angelin [1,2]

CONTENTS

SUPPLEMENTARY MATERIALS AND METHODS 3

Study design and oversight 3

Collection of IF and skin biopsies 4

Serum and IF lipoproteins 4

Ex vivo binding to human arterial proteoglycans. 5

Measurement of LDL aggregation susceptibility 5

LDL Lipid Mass Spectrometry 6

Skin tissue cholesterol 7

Sample size calculation 7

Assessment of normality and data transformation 7

Primary outcomes 8

Secondary outcomes 8

Multiple regression analysis 8

SUPPLEMENTARY FIGURES 9

Figure S1. Flow diagram of inclusion of T2D patients and controls 9

Figure S2. Graphic representation of observed relationships between age and interstitial-fluid-to-serum for albumin in controls and T2D in the Extended cohort 10

Figure S3. Influence of sex, age, medication, vascular complications, and diabetes control 11

SUPPLEMENTARY TABLES 12

Table S1. Demographic, clinical, and biological characteristics of T2D patients in the initial and newly recruited cohort 12

Table S2. Serum and IF proteins, lipoprotein lipids, and apolipoproteins in T2D patients and healthy controls in the initial and newly recruited cohort 13

Table S3. Univariate Pearson correlations between Interstitial-Fluid-to-serum ratios for albumin and apoB, LDL proteoglycan binding susceptibility, age, glycated and oxidized LDL with demographic, clinical, Serum, IF and IF:S variables in the extended cohort 14

Table S4. LDL Lipid Classes and Pairwise Statistical Comparisons across highest and lowest quartiles of IF:S for apoB in T2D patients and Controls 15

SUPPLEMENTARY REFERENCES 16

SUPPLEMENTARY MATERIALS AND METHODS

Study design and oversight

The collection of samples in the newly recruited cohort was performed at the clinical science center at Karolinska University Hospital Huddinge between 2018 and 2019, in the same facilities where the samples from the initial cohort was collected (1). Type 2 diabetic (T2D) patients included in the study were diagnosed according to WHO criteria(2). Exclusion criteria for T2D subjects and controls in the newly recruited cohort were in line with those for the Initial cohort; for patients with T2D: systemic inflammatory disease, thyroid disease, treatment with oral glucocorticoids, or levels of inflammatory markers, thyroid hormones, or haemoglobin outside laboratory reference range, pregnancy, systemic skin disease (not applicable for the Initial cohort); for healthy controls: blood pressure over 140/90, pregnancy, laboratory analyses outside laboratory reference range, or non-trivial disease requiring continuous systemic medical treatment, systemic skin disease (not applicable for the Initial cohort). The one notable difference in exclusion criteria between the Initial and newly recruited cohorts was that antihypertensive medication was accepted in controls in the newly recruited cohort, if not more than one antihypertensive drug and not including alpha-blockers, beta-blockers, or thiazide-diuretics (Figure S1).

Subjects arrived in the morning after an overnight fast. Height, weight, and bioimpedance measurements were registered. Blood pressure was measured in the sitting position. Phlebotomy was performed, blood was sent to the hospital central lab at Karolinska Hospital Huddinge for routine analysis of B-hemoglobin, P-bilirubin, P-ASAT, P-ALAT, P-ALP, S-TSH, S-T4, P-cystatin C, P-creatinine, P-hsCRP. Blood samples were also used for plasma and serum isolation and immediately frozen in aliquots at −80 °C.

The study was approved by the local ethics committee in Stockholm (Approval Number 2017-1942-31, 2018-1428-32). In a limited number of participants, the screening and study visit detected previously unknown diseases or risk factors. These participants were referred to the health service for further investigation and/or treatment. The study involved the use of the suction blister technique (3) for collecting interstitial fluid that has been previously used with good results and no adverse events (2). Prior to injection of local anaesthesia, the subjects were specifically asked for allergy or hypersensitivity to local anaesthetics. The study visit lasted circa 3 hours. The study was conducted with a focus on participant safety and in accordance with the Declaration of Helsinki (14).

Collection of IF and skin biopsies

The collection of serum and IF was performed in the same way as in the initial study (1). Subjects were placed in bed in the supine position. Suction blister fluid was generated ad modum Kiistala (3). Two plastic cups (Forma plast, Ockelbo, Sweden, not used in the Initial cohort, or Ventipress Oy, Lappeenranta, Finland) were placed circa 5 cm lateral on each side of the belly button on each subject. A mild negative pressure (28-32 kPa) was generated and sustained using a pump (Atmos S351, not used for the Initial cohort, or Ventipress Oy 3300 VAC). The 5 skin blisters generated under each cup contain suction blister fluid that is analogous to pre-nodal lymph and representative of IF. The IF was collected from the blisters with a syringe. The mean time to IF collection was 84 minutes (range, 45-143); mean volume of IF generated per subject was 225µl (range, 90-540). There was no significant difference in time to blister puncture or volume of IF collected between controls and T2D, and no differences between the different cups or pumps used. Mean time to blister formation and mean volume of IF generated did not differ between the Initial and newly recruited cohort and were not found to be confounding factors for any dependent variable of interest. One hour after the application of negative pressure under the cups, venous blood was drawn and stored as plasma, serum and whole blood at -80°C. Three regular 4-mm punch biopsies were taken from the belly skin lateral to where the suction cups had been from 39 diabetics and 39 controls, the procedure was performed under local anesthesia. Subcutaneous fat was separated from the dermis and epidermis with a knife under a microscope, and biopsies were snap frozen on dry ice.

Serum and IF lipoproteins

Frozen aliquots of samples from the initial and newly recruited cohorts were analyzed as an Extended cohort. Serum and IF lipoproteins were analyzed by fast performance liquid chromatography (FPLC) (4). Newly generated FPLC-data from the Initial cohort was highly correlated with previously reported data (1) (r=0.899-0.956), indicating no apparent effects of freezing or long-term storage. Lipoproteins from serum (2 µl) and IF (5 µl for cholesterol and 10 µl for TGs) were separated on a Superose 6 PC 3.2/300 column (GE Healthcare Bio-Sciences AB, Uppsala, Sweden) and eluted in fractions appearing in the exclusion volume of the sepharose column that contained chylomicrons (if present) together with VLDL and remnants (referred to as VLDL/remnants), then LDL, and last HDL. Reagents (CHOL2 ref 05168538, TRIGL ref 05171407 Cobas Roche Diagnostics, Mannheim, Germany) were continuously added to the eluate online, absorbance was measured at 512/659 nm and concentrations were calculated after integration of the individual chromatograms. LDL and HDL cholesterol particle sizes were estimated by interpolation of retention times in a linear regression model (5) using retention times for LDL and HDL in healthy controls (being 39.3 and 49.8 minutes, respectively, together with published particle sizes in healthy subjects (LDL 21.1 nm, and HDL 8.9 nm).(6, 7) Apolipoproteins were analyzed by ELISAs (Mabtech, Nacka strand, Sweden) following manufacturer's instructions. Albumin

was assayed with BCG Albumin Assay Kit (Sigma-Aldrich, Saint Louis, MO, USA). Glycated LDL was assayed with ELISA (LSBio, Seattle, WA, USA) and reported as percent glycated LDL. Oxidized LDL was assayed by ELISA (Mercodia,Uppsala, Sweden) and reported as proportion of oxidized LDL by dividing OX-LDL (U/L) with LDL cholesterol (mmol/L). Oxidized LDL was measured using a 4E6-based assay directed against oxidatively modified apoB epitopes, rather than an E06-based oxPL assay.

Ex vivo binding to human arterial proteoglycans.

Serum lipoproteins for *ex vivo* assays were isolated by sequential ultracentrifugation (8, 9). Determinations of whole serum lipoprotein proteoglycan binding susceptibility (S-LPBS), LDL-proteoglycan binding susceptibility (LDL-PBS) and VLDL-proteoglycan binding susceptibility (VLDL-PBS) were performed using a solid-phase binding assay that has been found reproducible for evaluation of relative differences in the affinity of isolated, or serum apoB-lipoproteins for human arterial proteoglycans (10, 11). In brief, polystyrene 96-well plates were coated with 100 µl aortic proteoglycans isolated from the intima media of human aortas (12) (50 µg/ml glycosaminoglycan in phosphate buffered saline) by incubation at 4°C overnight. Wells were blocked with 1% BSA in phosphate buffered saline for 1 h at 37°C following addition of either 1 µL of plasma, 5µg of VLDL/remnants or 2µg of LDL in 140 mmol/L NaCl, 2 mmol/L $MgCl_2$, 5 mmol/L $CaCl_2$, 10 mmol/L MES, pH 5.5, and incubated for 1 h at 37°C. The wells were washed with 10 mmol/L MES-50 mmol/L NaCl, pH 5.5, and the amount of bound cholesterol was determined using the Amplex Red cholesterol kit (Thermo Fisher Scientific, BV Europe). Each sample was analyzed in duplicate and to correct for non-specific binding, the cholesterol bound in a non-coated well was subtracted from each sample. To correct for interindividual differences in levels of lipoproteins in each individual sample, results are expressed as bound cholesterol adjusted for total serum cholesterol (nmol/TC) for serum and for µg protein (nmol/µg protein) for VLDL/remnants and LDL.

Measurement of LDL aggregation susceptibility

The measurement of LDL aggregation susceptibility was performed as previously described (13, 14) except serum, rather than plasma, was used. To validate this approach, we compared LDL aggregation susceptibility between serum and plasma samples from a subset of donors and found no significant differences (data not shown). Particle sizes of isolated LDL (200 µg/mL in 20 mM MES, pH 5.5, containing 150 mM NaCl and 50 µM $ZnCl_2$) were measured at baseline (0h) using dynamic light scattering (Wyatt DynaPro Plate Reader II; Wyatt Technology, CA). Aggregation was induced with human recombinant sphingomyelinase and particle aggregation was followed by measuring the aggregate size at approximately every 15 minutes for 6 hours. Aggregation data were collected with Dynamics V7 software (Wyatt Technology, CA).

LDL Lipid Mass Spectrometry

Mass spectrometry (MS)-based lipidomics analysis of isolated LDL was conducted on a subset of individuals from the extended cohort. Subjects were ranked according to their interstitial fluid to serum (IF:S) ratio for ApoB. From the lowest quartile, 10 control subjects and 8 T2D patients were randomly selected, while from the highest quartile, 10 control subjects and 10 T2D patients were randomly selected. Samples were processed using a two-phase chloroform/methanol extraction (15). First, 10 µg isolated LDL was diluted into 0.2 ml of ammonium formate (0.2 M) and 5 µl of the internal SPLASH lipidomix standard mix and 10µl of ceramide (Cer) 18:1;O2/12:0 (Sigma-Aldrich, Merck Life Science, Espoo, Finland) were added as internal standards. A volume of 0.75 ml chloroform-methanol 1:2 (CM 1:2, v/v) was then added and samples were mixed with a ThermoMixer (Eppendorf, Hamburg, Germany) for 15 min at 1400 rpm at RT. A volume of 0.25 ml of 0.2 M ammonium formate and 0.25 ml chloroform was added and the samples were further mixed for 15 min at 1400rpm at RT. Phase separation was achieved after centrifugation for 5 min at 5000 x g at +4 °C. The lower phase was collected, and the remaining upper phase was re-extracted by adding 0.5 ml of chloroform and mixing for 15 min at 1400 rpm at RT followed by a centrifugation for 5 min at 5000 x g at +4 °C. The new lower phase was collected and combined with the previous lower phase before evaporation by vacuum evaporator. The evaporated extracts were reconstituted in 0.25 ml of CM 1:2, transferred to 1.5 ml borosilicate glass sample vials, capped and stored at – 20 °C until analysis.

The extracted LDL samples were analyzed using an LC/MS system as shown before (16). A volume of 5 µl of each sample was injected into the Agilent 1290 Infinity HPLC system (Santa Clara, CA, USA) using an autosampler. Chromatographic separation was conducted in a gradient mode with a Luna Omega C18 100 Å (50 × 2.1 mm, 1.6 µm) column (Phenomenex, Torrance, CA, USA), and employing an acetonitrile/water/isopropanol-based solvent system (17) with the flow rate of 0.200 ml/min and 25 °C as the column temperature. The column eluent was infused into the electrospray source of an Agilent 6490 Triple Quad LC/MS (Santa Clara, CA, USA) with iFunnel Technology and spectra were recorded in positive ionization modes. MS+ scan was used to detect neutral TG lipids as NH4 adducts. Specific precursor ion scans were used for PC, LPC, SM (precursor of m/z 184), Cer and HexCer (precursor of m/z 264), and CE (precursor of m/z 369) species. Spectra were extracted from the chromatogram with Agilent MassHunter Qualitative Navigator v B.08.00 according to known elution time windows for all identified lipid classes and individual lipid species in each class were identified and quantified using LIMSA software on Excel according to Haimi et al.(18). Concentration values for each individual lipid species were calculated as pmol/µg of LDL. Data were also converted into molar% and individual lipid

species below 0.5% were excluded. Lipid class profiles were created by summing all the individual lipid species in the same lipid class together.

Skin tissue cholesterol

Lipids were extracted from biopsies using 5mL chloroform:methanol (2:1 vol/vol) at +60°C overnight and the extract dried under nitrogen gas. Samples were resuspended in 2.5mL hexane and divided into two aliquots of 250uL, containing 1000 ng D7 cholesterol (Steraloids, Inc. Newport, RI, USA). One aliquot was dried under nitrogen for free cholesterol analysis. KOH (0.5 M) in ethanol was added to the other aliquot and heated to 60°C in a water bath for 1h where water and hexane were added before centrifugation at 3.000 g for 5 min. The upper phase was dried under nitrogen for total cholesterol analysis. All samples were then silylated with 0.4 ml of pyridine/hexametyl-disilizane/chlorotrimetylsilane (3:2:1, v/v/v) at 60°C for 30min, and dried under nitrogen at 60°C. Samples were dissolved in 100 µL of hexane and analyzed using GC/MS. The amount of esterified cholesterol was calculated by subtracting the free cholesterol from total cholesterol and results are expressed as ug cholesterol adjusted for dry weight of biopsies post lipid extraction (ug/mg d.w).

SUPPLEMENTARY STATISTICAL METHODS

Sample size calculation

The initial study aimed to detect a 25% increase in IF-to-serum ratio of LDL-cholesterol in T2D patients with 95% power at a significance level of 0.05 (1). The primary outcome measure used for calculating power for the newly recruited cohort in the present, extended study, was the difference in IF-to-serum ratio of LDL-cholesterol and apoB between T2D subjects and matched controls in the initial study. The number of subjects was calculated to give 80% power to detect a difference at a significance level of 0.05. We used STATAMP 15.1 (StataCorp 4905 Lakeway Drive College Station, Texas 77845 USA) for power calculations.

Assessment of normality and data transformation

Normality of all continuous variables was assessed using the Shapiro–Wilk test and visual inspection of Q–Q plots. Given the large sample size (n=74 per group), minor deviations from normality were considered acceptable for parametric analysis if visual inspection indicated an approximately normal distribution, even when the Shapiro–Wilk test was significant. Serum and IF triglycerides showed marked skewness by both the Shapiro–Wilk test and Q–Q plots, and were log-transformed prior to analysis.

Primary outcomes

- Differences in levels and distribution of albumin, cholesterol, TG, and apolipoproteins in serum and IF, as well as IF-to-serum ratios of these variables in T2D subjects and controls between the initial and newly recruited cohorts.
- Differences in levels and distribution of albumin, cholesterol, TG, and apoproteins in serum, as well as IF-to-serum ratios of these variables IF between T2D subjects and controls in the extended cohort.
- Differences in serum lipoprotein proteoglycan binding susceptibility and LDL aggregation susceptibility between T2D subjects and controls in the extended cohort.
- Differences in the correlation between serum lipoprotein proteoglycan binding susceptibility and LDL aggregation susceptibility and IF-to-serum ratios of albumin, cholesterol, TG, and apoproteins between T2D subjects and controls in the extended cohort.
- Differences in levels of total, free and esterified cholesterol in skin between T2D subjects and controls in a subgroup of the newly recruited cohort.

Secondary outcomes

- The influence of sex, age-groups, statin use, metformin use, macrovascular complications, microvascular complications, cystatin c levels and HbA1c levels on levels and distribution of albumin, cholesterol, TG and apoproteins in serum, IF, skin and IF-to-serum ratios of these variables in the extended cohort.
- The influence of sex, age-groups, statin use, metformin use, macrovascular complications, microvascular complications, cystatin c levels and HbA1c levels on serum lipoprotein proteoglycan binding susceptibility and LDL aggregation susceptibility in the extended cohort.

  The same methods of analysis used for the primary outcomes were used for the secondary outcomes.

Multiple regression analysis

Parameters were selected based on their clinical relevance and statistical significance in univariate analysis. Model fit was evaluated by residual plots (data not shown), R-squared and adjusted R-squared, Akaike´s information criterion (AIC) and Bayesian information criterion (BIC). The parameters HbA1c, time since T2D diagnosis, sex, systolic blood pressure, cystatin c, hsCRP, statin treatment and serum non-HDL cholesterol were excluded from the final multiple regression model as they were not statistically significant in univariate analysis ($p > 0.05$) and did not improve model fit, as assessed by AIC, BIC, or adjusted R^2.BMI, Glycated G-LDL %, and OX-LDL/LDLc showed no independent associations

with the apoB ratio in multivariable models, and did they did not materially alter the effect estimate for T2D. The model provides a strong overall fit, explaining about 60% of the variance in ratioApoBgL, and 59% when adjusted for the number of independent parameters. The residuals are approximately normally distributed (data not shown). The AIC and BIC values suggest that there is good balance between complexity and fit in this model. Multicollinearity was checked and found not to be an issue showing low variance inflation factors (VIFs) throughout. One of the key clinical observations in our study is the differential effect of age on apob ratio in controls compared with T2D subjects. This regression model was unable to effectively capture this interaction due to very high multicollinearity when an age × T2D interaction term was introduced. The issue persisted even after attempts to mitigate multicollinearity through standardization and other transformations.

SUPPLEMENTARY FIGURES

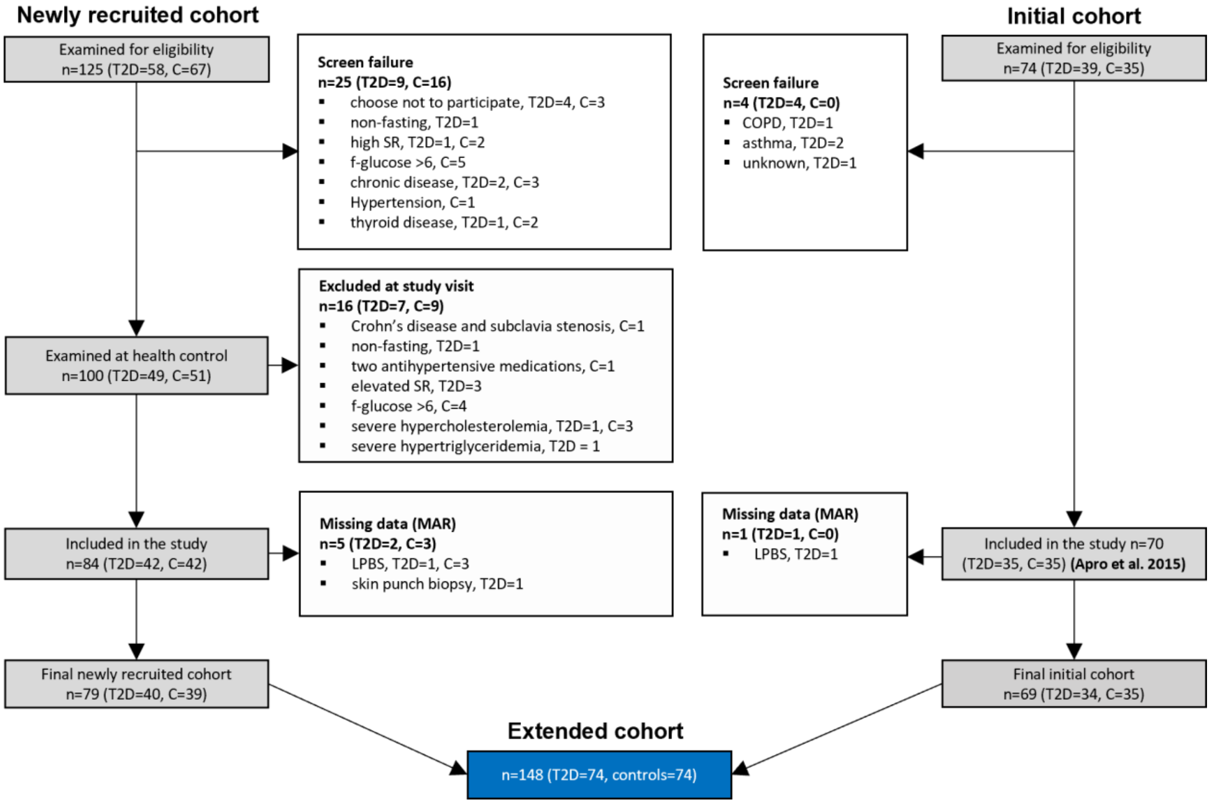

Figure S1. Flow diagram of inclusion of T2D patients and controls

COPD; chronic obstructive pulmonary disease, SR, sedimentation rate; MAR, missing at random; LPBS, lipoprotein proteoglycan binding susceptibility.

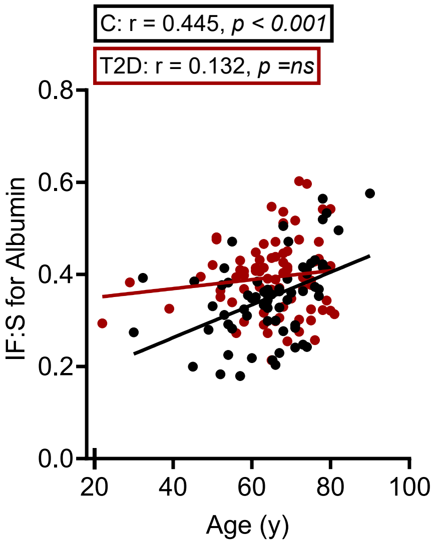

Figure S2. Graphic representation of observed relationships between age and interstitial-fluid-to-serum for albumin in controls and T2D in the Extended cohort

n= 74:74 (C:T2D). Pearson correlation coefficients were obtained from univariate regression analyses. p< 0.05 indicates statistical significance. IF:S, Interstitial Fluid-to-Serum ratio

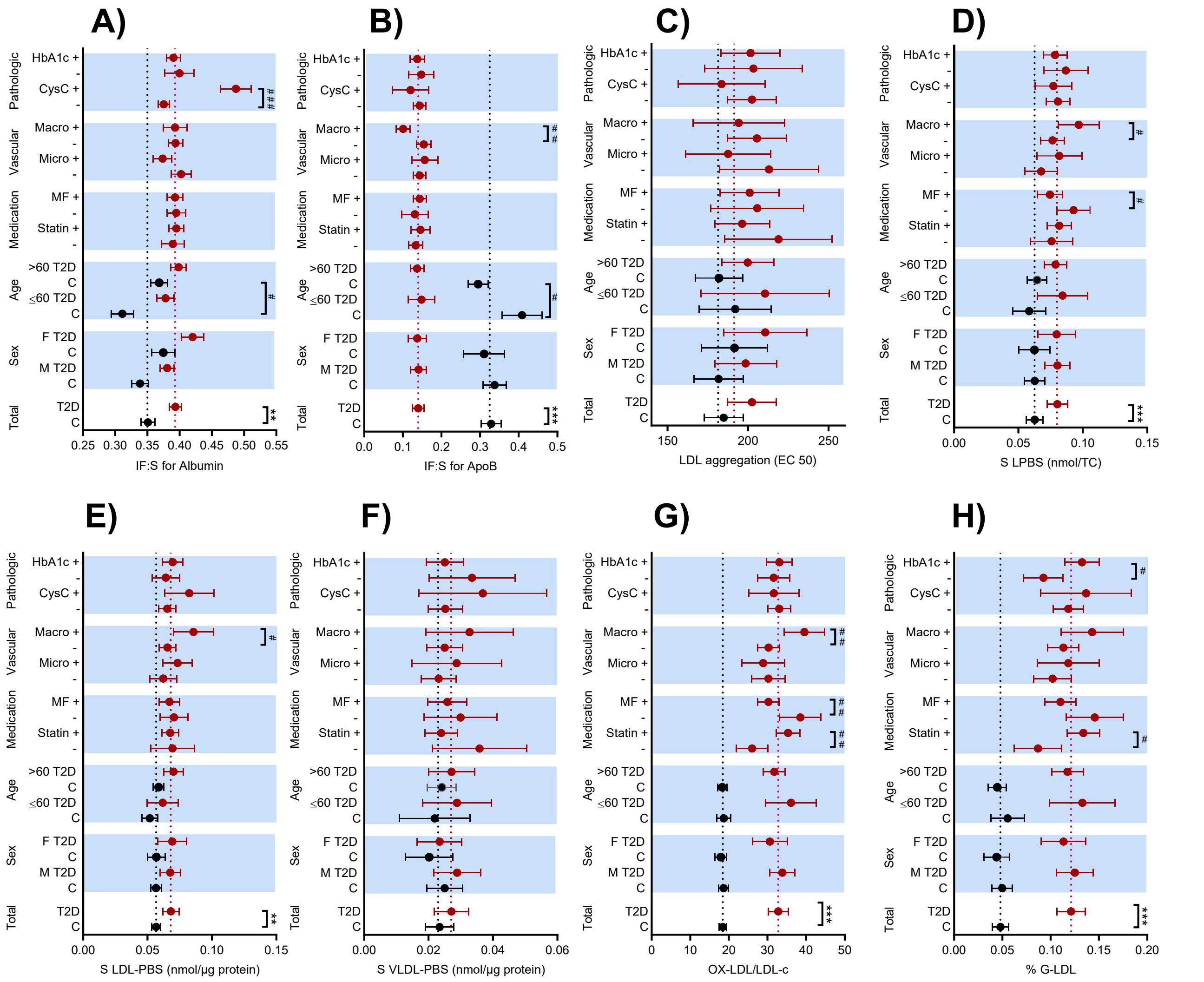

Figure S3. Influence of sex, age, medication, vascular complications, and diabetes control *A) IF:S albumin B) IF:S apoB C) LDL aggregation D-F) lipoprotein proteoglycan binding susceptibility. G) Proportion of oxidized LDL cholesterol H) Percent glycated LDL cholesterol. Total nr of subjects 74:74 (C:T2D); Sex 50:24 (M:F); Age group <60:>60 22:52(C), 18:56 (T2D); MF 51:23 (+/-); Statins 54:20 (+/-); Vascular Micro only:no complications 17:25 (+/-); Vascular Macro:no macro complications 20:54 (+/-); Pathological CysC 12:62 (+/-); Pathological HbA1c 54:19 (+/-). Data presented as mean ± 95%CI. Dotted lines represent mean value of controls and T2D. Significant levels for differences between C and T2D: *p<0.05 **p<0.01, ***p<0.001, student's t-test; significance levels for differences in subgroups: #p<0.05 ##p<0.01, ###p<0.001, one way ANOVA with Benjamini and Hochberg false discovery rate (FDR) correction. IF:S, Interstitial Fluid-to-Serum ratio; LPBS, Lipoprotein proteoglycan binding susceptibility; %G-LDL, percent glycated LDL cholesterol. OX-LDL/LDLc, oxidized LDL adjusted for LDL cholesterol. MF, metformin; Vascular-micro, diabetic microvascular complications; Vascular-macro, major adverse cardiovascular event; CysC, cystatin C.*

SUPPLEMENTARY TABLES

Table S1. Demographic, clinical, and biological characteristics of T2D patients in the initial and newly recruited cohort

	Initial cohort #			*Newly recruited cohort*			*Initial vs Newly recruited T2D*
	C	T2D	*p*	C	T2D	*p*	*p*
Males : Females (n:n)	28:7	26:8		22:17	24:16		
Age (y)	60.8 ± 9.6	60.5 ± 12.1	NS	68.9 ± 10.5	68.7 ± 8.2	NS	<0.001
BMI (kg/m2)	25.4 ± 3.1	31.7 ± 5.2	<0.001	25.1 ± 2.7	28.2 ± 4.2	<0.001	<0.01
HbA1c (mmol/mol)	37.3 ± 3.5	63.6 ± 18.6	<0.001	37.0 ± 3.8	54.1 ± 12.2	<0.001	<0.01
FPG (mM)	5.1 ± 0.4	8.5 ± 3.1	<0.001	5.3 ± 0.3	7.3 ± 1.4	<0.001	0.03
SBP (mmHg)	128.1 ± 9.7	145.8 ± 14.6	<0.001	130.8 ± 14.0	137.6 ± 15.8	<0.001	0.02
CysC (mg/l)	0.86 ± 0.11	1.04 ± 0.47	0.04	0.94 ± 0.14	1.06 ± 0.24	0.04	NS
hsCRP (mg/l)	1.99 ± 2.92	2.42 ± 2.34	NS	1.16 ± 1.14	1.68 ± 2.39	NS	NS
Diabetes duration (y)	-	13.8 ± 8.1		-	8.5 ± 7.9		<0.01
AD:s (%)	-	100		-	83		<0.01
LLT (%)	-	70		5	78		NS
Prior MACE (%)	-	38		-	18		0.04
Pathological CystC (%)	-	18		5	15		NS
Pathological HbA1c (%)	-	76		-	70		NS

AD, antidiabetic drug treatment; BMI, body mass index; CysC,, Cystatin C; DBP, diastolic blood pressure; FBG, fasting blood glucose; HbA1c, glycosylated hemoglobin; hsCRP, high-sensitivity C-reactive protein; LLT, lipid lowering therapy; MACE, major cardiovascular event; NS, not significant; SBP, systolic blood pressure; Pathological HbA1c >48 mmol/mol; Pathological CysC >1.25 mg/L. Data presented as mean ± SD and percentages. One value for HbA1c in a T2D patient is missing. One value for hsCRP in a control is missing. # Ref(1). Unadjusted pairwise comparisons between two independent groups were conducted using Student's t-test.

Table S2. Serum and IF proteins, lipoprotein lipids, and apolipoproteins in T2D patients and healthy controls in the initial and newly recruited cohort

			Initial cohort			*Newly recruited cohort*		
			C	T2D	*p*	C	T2D	*p*
Serum		Albumin	39.47 ± 2.38	39.8 ± 3.22	<.01	38.23 ± 2.37	40.46 ± 3.15	<.01
	Chol (mmol/L)	Total	5.45 ± 1.04	3.91 ± 1.24	<.001	5.74 ± 0.81	4.00 ± 0.73	<.001
		VLDL	0.52 ± 0.37	0.63 ± 0.39	NS	0.28 ± 0.24	0.40 ± 0.099	NS
		LDL	3.42 ± 0.89	2.40 ± 0.98	<.001	3.79 ± 0.79	2.45 ± 0.11	<.001
		HDL	1.50 ± 0.43	0.89 ± 0.28	<.001	1.71 ± 0.48	1.26 ± 0.054	<.001
		non - HDL	3.95 ± 1.10	3.02 ± 1.16	<.001	4.03 ± 0.88	2.73 ± 0.82	<.001
	TG (mmol/L) †	Total	1.40 ± 0.71	2.40 ± 1.33	<.001	0.98 ± 0.54	1.73 ± 0.88	<.001
		VLDL	0.77 ± 0.66	1.79 ± 1.37	<.001	0.43 ± 0.41	1.19 ± 0.86	<.001
		LDL	0.44 ± 0.16	0.44 ± 0.14	NS	0.41 ± 0.17	0.38 ± 0.14	NS
		HDL	0.18 ± 0.06	0.17 ± 0.05	NS	0.13 ± 0.06	0.15 ± 0.06	NS
	Apos	ApoB (g/L)	0.76 ± 0.25	0.85 ± 0.24	NS	0.78 ± 0.25	0.80 ± 0.22	NS
		Apo E (mg/L)	25.2 ± 9.7	30.42 ± 10.0	NS	18.8 ± 11	20.5 ± 7	NS
IF		Albumin	13.29 ± 3.06	15.65 ± 3.38	<.05	13.73 ± 3.14	15.88 ± 3.24	<.05
	Chol (mmol/L)	Total	1.08 ± 0.35	0.64 ± 0.24	<.001	0.97 ± 0.35	0.59 ± 0.22	<.001
		VLDL	0.06 ± 0.04	0.04 ± 0.03	NS	0.02 ± 0.01	0.01 ± 0.01	NS
		LDL	0.61 ± 0.24	0.34 ± 0.16	<.001	0.51 ± 0.22	0.25 ± 0.13	<.001
		HDL	0.40 ± 0.14	0.25 ± 0.10	<.05	0.45 ± 0.17	0.33 ± 0.14	<.05
		non - HDL	0.67 ± 0.26	0.38 ± 0.17	<.001	0.52 ± 0.22	0.25 ± 0.14	<.001
	TG (mmol/L) †	Total	0.22 ± 0.11	0.21 ± 0.11	NS	0.16 ± 0.07	0.17 ± 0.12	NS
		VLDL	0.09 ± 0.07	0.11 ± 0.10	<.05	0.03 ± 0.02	0.07 ± 0.05	<.05
		LDL	0.08 ± 0.03	0.06 ± 0.02	NS	0.06 ± 0.02	0.06 ± 0.04	NS
		HDL	0.06 ± 0.02	0.05 ± 0.02	NS	0.03 ± 0.01	0.04 ± 0.03	NS
	Apos	ApoB (g/l)	0.27 ± 0.10	0.12 ± 0.06	<.001	0.22 ± 0.08	0.15 ± 0.07	<.001
		Apo E (mg/L)	4.0 ± 1.6	3.1 ± 1.1	NS	3.0 ± 1.9	2.8 ± 1.3	NS
IF: S		Albumin	0.34 ± 0.08	0.39 ± 0.08	<.01	0.36 ± 0.09	0.39 ± 0.08	<.01
	Chol (mmol/L)	Total	0.19 ± 0.06	0.17 ± 0.07	NS	0.17 ± 0.058	0.15 ± 0.05	NS
		VLDL	0.11 ± 0.05	0.08 ± 0.07	NS	0.07 ± 0.06	0.070 ± 0.08	NS
		LDL	0.18 ± 0.06	0.14 ± 0.06	<.01	0.13 ± 0.051	0.10 ± 0.04	<.01
		HDL	0.27 ± 0.08	0.29 ± 0.10	NS	0.27 ± 0.090	0.27 ± 0.08	NS
		non - HDL	0.17 ± 0.06	0.13 ± 0.06	<.01	0.13 ± 0.049	0.10 ± 0.04	<.01
	TG (mmol/L) †	Total	0.17 ± 0.07	0.11 ± 0.05	<.001	0.18 ± 0.10	0.11 ± 0.08	<.001
		VLDL	0.11 ± 0.06	0.07 ± 0.04	<.05	0.10 ± 0.08	0.07 ± 0.05	<.05
		LDL	0.20 ± 0.09	0.15 ± 0.06	<.05	0.15 ± 0.09	0.12 ± 0.08	<.05
		HDL	0.32 ± 0.12	0.27 ± 0.08	NS	0.31 ± 014	0.30 ± 0.10	NS
	Apo's	ApoB (g/l)	0.37 ± 0.11	0.12 ± 0.06	<.001	0.29 ± 0.10	0.16 ± 0.07	<.001
		Apo E (mg/L)	0.16 ± 0.06	0.11 ± 0.03	NS	0.18 ± 0.14	0.16 ± 0.11	<.05

Apos, Apolipoproteins; Chol, Cholesterol; IF, Interstitial Fluid; IF:S, Interstitial Fluid-to-Serum ratio; NS, not significant; TG, Triglycerides; Data presented as mean ± SD. † Logarithmic transformed before t-test.# Ref(1).

Table S3. Univariate Pearson correlations between Interstitial-Fluid-to-serum ratios for albumin and apoB, LDL proteoglycan binding susceptibility, age, glycated and oxidized LDL with demographic, clinical, Serum, IF and IF:S variables in the extended cohort

		**IF:S Albumin**		**IF:S ApoB**		**LDL-PBS**		**Age**		**G-LDL**		OX-LDL	
		C	T2D	C	T2D	C	T2D	C	T2D	C	T2D	C	T2D
	Age	**0.445*** **	0.132	**-0.395*** **	-0.066	**0.428*** **	0.094	1	1	-0,105	**-0,245***	0,009	-0,121
	BMI	0.057	**0.318 ****	**-0.300 ****	-0.126	0.094	0.200	-0.016	-0.17	0,015	0,213	0,076	-0,023
	SBP	**0.322****	0.031	**-0.240 ***	-0.16	**0.281***	0.028	**0.424*** **	0.134	-0,02	0,028	0,026	-0,072
	FPG	0.192	-0.017	**-0.396*** **	-0.028	**0.377****	0.019	**0.411*** **	**-0.232***	-0,116	**0,503*** **	-0,006	0,182
	Insulin	-0.083	0.165	0.155	0.066	-0.184	-0.080	**-0.241***	-0.145	-0,145	0,133	-0,013	0,132
	HbA1c	0.046	0.145	-0.014	-0.147	0.125	0.124	**0.359****	-0.156	0,015	0,568	0,038	0,079
	CysC	0.195	**0.457 *** **	**-0.247 ***	-0.098	0.078	0.200	**0.385*** **	**0.285***	-0,127	0,087	-0,105	0,045
S	Albumin	**-0.42*** **	-0.094	**0.300***	0.094	**-0.372****	0.065	**-0.701*** **	**-0.275***	0,109	-0,001	0,007	0,060
	Total Chol	-0.022	0.111	0.004	0.098	**0.24***	0.134	**0.302****	0.082	**-0,303 ****	**-0,258***	**-0,581*** **	**-0,706*** **
	VLDL-c	0.086	0.105	-0.030	-0.078	0.075	0.117	-0.103	-0.034	-0,195	0,115	-0,185	-0,050
	LDL-c	0.021	0.111	-0.103	0.125	**0.314****	0.177	**0.334****	0.037	-0,196	-0,205	**-0,605*** **	**-0,759*** **
	G-LDL	-0,085	-0,088	0,034	**-0,322****	**-0.23***	**0.365****	-0,105	**-0,245***	1	1	0,016	-0,128
	OX-LDL	0,025	-0,072	-0,049	**-0,27***	0.04	0.08	0,009	-0,121	0,193	**0,443*** **	0,192	**0,444*** **
	HDL-c	-0.187	-0.184	0.158	0.118	-0.138	-0.051	0.089	0.160	0,009	-0,127	1	1
	Total TG †	0.057	**0.241** *	0.023	-0.023	-0.014	0.088	0.019	-0.113	-0,178	**0,27***	-0,162	0,188
	VLDL-TG †	0.105	0.202	0.034	0.040	0.07	0.101	-0.04	-0.096	-0,174	**0,256***	-0,121	0,210
	LDL-TG †	0.017	0.214	-0.011	**-0.301****	-0.06	0.040	**0.226***	-0.032	-0,086	0,214	-0,225	-0,0526
	HDL-TG †	-0.153	0.03	0.177	-0.004	**-0.223***	-0.117	-0.109	-0.089	-0,060	0,107	0,009	-0,023
	ApoB	0.107	0.109	**-0.356***	0.119	**0.420*** **	0.080	0.134	-0.124	-0,226	-0,033	**-0,301****	**-0,496*** **
	ApoE	-0.076	-0.032	0.031	-0.144	**-0.208***	0.03	0.003	-0.083	-0,141	0,247*	-0,107	**0,256***
IF	Albumin	**0.965***	**0.915 *** **	-0.081	-0.024	0.193	-0.038	**0.272***	0.029	-0,060	-0,084	0,013	-0,040
	Total Chol	-0.161	0.033	**0.621*** **	0.19	**-0.290***	-0.18	-0.161	-0.185	-0,138	**-0,25***	-0,135	-0,254*
	VLDL-c	0.004	0.009	**0.304****	0.138	**-0.220***	-0.173	**-0.239***	-0.173	-0,116	0,146	-0,024	0,157
	LDL-c	-0.073	0.06	**0.533*** **	0.185	**-0.210***	-0.071	-0.119	**-0.303****	-0,171	-0,151	-0,210	**-0,323****
	HDL-c	**-0.263***	-0.041	**0.514*** **	0.066	-0.059	-0.06	-0.122	0.079	-0,021	-0,212	0,002	0,029
	Total TG †	0.214	0.085	**0.454*** **	-0.152	**-0.29***	0.056	-0.207	-0.091	-0,077	0,013	-0,076	0,037
	VLDL-TG †	0.175	0.074	**0.397*** **	-0.073	-0.185	0.072	-0.217	-0.013	-0,127	0,070	-0,001	0,108
	LDL-TG †	0.07	0.107	**0.482*** **	-0.09	**-0.420*** **	0.018	-0.165	-0.119	-0,044	-0,069	-0,062	-0,096
	HDL-TG †	0.079	0.101	**0.472*** **	**-0.290***	**-0.340****	0.070	**-0.300***	-0.067	0,046	0,013	0,035	0,059
	ApoB	-0.08	0.034	**0.584*** **	**0.352****	-0.094	0.147	-0.228	-0.129	-0,117	**-0,290***	**-0,236***	**-0,430*** **
	ApoE	-0.159	-0.167	**0.236***	0.136	**-0.252***	0.143	**-0.242***	-0.079	0,074	0,098	0,025	-0,035
IF:S	Albumin	1	1	-0.154	-0.056	**0.310****	-0.076	**0.449*** **	0.132	-0,085	-0,088	0,025	-0,072
	Total Chol	-0.153	-0.071	**0.693*** **	0.109	**-0.303****	**-0.232***	**-0.362****	**-0.267***	0,006	-0,185	0,131	-0,036
	VLDL-c	-0.094	-0.192	**0.482*** **	0.179	**-0.311****	-0.23	-0.131	0.036	0,116	-0,198	**0,269***	-0,149
	LDL-c	-0.09	-0.029	**0.684*** **	0.122	**-0.295***	**-0.204***	**-0.382*** **	**-0.424****	-0,027	-0,16ä6	0,144	-0,083
	HDL-c	-0.12	0.103	**0.446*** **	-0.01	-0.035	-0.019	-0.209	-0.118	-0,061	-0,134	-0,022	0,085
	Total TG	0.106	-0.063	**0.460*** **	-0.03	-0.205	-0.069	**-0.27 ***	0.101	0,058	-0,106	0,092	0,058
	VLDL-TG	0.109	-0.11	**0.479*** **	-0.028	-0.008	-0.070	-0.251*	0.166	-0,048	-0,070	0,092	0,093
	LDL-TG	-0.029	0.102	**0.433*** **	0.101	**-0.295****	0.033	**-0.39*** **	0.049	0,062	-0,12	0,148	-0,032
	HDL-TG	0.080	0.066	0.123	-0.060	-0.133	0.187	-0.089	0.078	0,062	0,006	0,083	0,034
	ApoB	-0.157	-0.054	1	1	**-0.490*** **	**-0.379 ****	**-0.395*** **	-0.074	0,034	**-0,322****	-0,049	**-0,27***
	ApoE	-0.08	-0.055	-0.055	0.036	0.012	0.169	0.026	-0.01	**0,241***	-0,072	0,122	-0,064

G-LDL, percent glycated LDL cholesterol; OX-LDL, marker of oxidized LDL particles adjusted for serum LDL cholesterol, BMI, body mass index; CysC, Cystatin C; DBP, diastolic blood pressure; FBG, fasting blood glucose; HbA1c, glycosylated hemoglobin; SBP, systolic blood pressure. *p<0.05, **p<0.01, ***p<0.001. Grey indicates non-significant correlations. † Logarithmically transformed

Table S4. LDL Lipid Classes and Pairwise Statistical Comparisons across highest and lowest quartiles of IF:S for apoB in T2D patients and Controls

	Q1 C	Q4 C	Q1 T2D	Q4 T2D	Q1:Q4 (T2D:T2D)	Q1:Q4 (C:C)	Q1:Q1 (C:T2D)	Q4:Q4 (C:T2D)	Q4:Q1 (C:T2D)
n	10	10	8	10					
IF:S apoB, mean [range]	0.18 [0.10-0.24]	0.51 [0.3-0.71]	0.06 [0.04-0.08]	0.27 [0.2-0.36]	<0.001	<0.001	<0.01	<0.001	<0.05
Total lipid (pmol/µg)	13090 ± 1838	14110 ± 2641	10392 ± 2322	11633 ± 2284	ns	ns	< 0.01	ns	< 0.01
Core lipid (%)	85.4 ± 0.70	84.8 ± 1.80	85.3 ± 1.9	84.4 ± 2.00	ns	ns	ns	ns	ns
Surface lipid (%)	14.5 ± 0.70	15.1 ± 1.80	14.5 ± 1.9	15.5 ± 2.00	ns	ns	ns	ns	ns
CE (%)	75.1 ± 2.55	73.9 ± 3.35	62.6 ± 7.24	70.1 ± 4.50	< 0.01	ns	< 0.001	ns	< 0.001
TG (%)	10.3 ± 2.15	10.8 ± 3.27	22.69 ± 7.73	14.3 ± 4.76	< 0.01	ns	< 0.001	ns	< 0.001
Cer (%)	0.08 ± 0.03	0.08 ± 0.02	0.11 ± 0.04	0.12 ± 0.03	< 0.05	ns	ns	< 0.01	< 0.01
HexCer (%)	0.01 ± 0.00	0.02 ± 0.01	0.01 ± 0.01	0.01 ± 0.01	ns	ns	ns	ns	ns
LPC (%)	0.27 ± 0.04	0.32 ± 0.05	0.26 ± 0.03	0.28 ± 0.07	ns	ns	ns	ns	ns
PC (%)	10.5 ± 0.65	10.7 ± 1.54	10.9 ± 1.42	11.4 ± 1.50	ns	ns	ns	ns	ns
SM (%)	3.68 ± 0.64	4.12 ± 0.68	3.40 ± 0.60	3.77 ± 0.92	ns	ns	ns	ns	ns

Q1, Quartile 1; Q4, Quartile 4; C, Control; T2D;Type 2 Diabetes IF:S, Interstitial Fluid-to-Serum ratio; Cer, ceramide; HexCer, hexosylceramide; LPC, lysophosphatidylcholine; PC, phosphatidylcholine; SM, sphingomyelin; CE, cholesteryl ester; TG, triacylglycerol. Q1:Q4 (C): Comparison of Quartile 1 vs. Quartile 4 within Controls. Q1:Q4 (T2D): Comparison of Quartile 1 vs. Quartile 4 within T2D, Q1 C : Q1 T2D: Comparison of Quartile 1 between Control and T2D, Q4 C : Q4 T2D: Comparison of Quartile 4 between Control and T2D. Data presented as mean ± SD. Statistical significance was determined using one-way ANOVA followed by Tukey's post hoc test.

SUPPLEMENTARY REFERENCES

1. Apro J, Parini P, Broijersen A, Angelin B, and Rudling M. Levels of atherogenic lipoproteins are unexpectedly reduced in interstitial fluid from type 2 diabetes patients. *J Lipid Res.* 2015;56(8):1633-9.
2. . World Health Organization (2019). Classification of diabetes mellitus. Available from: https://www.who.int/publications/i/item/classification-of-diabetes-mellitus. Accessed August 20, 2025.
3. Kiistala U. Suction blister device for separation of viable epidermis from dermis. *J Invest Dermatol.* 1968;50(2):129-37.
4. Parini P, Johansson L, Bröijersén A, Angelin B, and Rudling M. Lipoprotein profiles in plasma and interstitial fluid analyzed with an automated gel-filtration system. *European journal of clinical investigation.* 2006;36(2):98-104.
5. Apro J, Tietge UJ, Dikkers A, Parini P, Angelin B, and Rudling M. Impaired cholesterol efflux capacity of high-density lipoprotein isolated from interstitial fluid in type 2 diabetes mellitus-Brief Report. *Arterioscler Thromb Vasc Biol.* 2016;36(5):787-91.
6. El Harchaoui K, van der Steeg WA, Stroes ES, Kuivenhoven JA, Otvos JD, Wareham NJ, et al. Value of low-density lipoprotein particle number and size as predictors of coronary artery disease in apparently healthy men and women: the EPIC-Norfolk Prospective Population Study. *J Am Coll Cardiol.* 2007;49(5):547-53.
7. El Harchaoui K, Arsenault BJ, Franssen R, Després JP, Hovingh GK, Stroes ES, et al. High-density lipoprotein particle size and concentration and coronary risk. *Ann Intern Med.* 2009;150(2):84-93.
8. Hallberg C, Hådén M, Bergström M, Hanson G, Pettersson K, Westerlund C, et al. Lipoprotein fractionation in deuterium oxide gradients: a procedure for evaluation of antioxidant binding and susceptibility to oxidation. *J Lipid Res.* 1994;35(1):1-9.
9. Äikäs L, Kovanen PT, Lorey MB, Laaksonen R, Holopainen M, Ruhanen H, et al. Icosapent ethyl-induced lipoprotein remodeling and its impact on cardiovascular disease risk markers in normolipidemic individuals. *JCI Insight.* 2025;10(19).
10. Sneck M, Kovanen PT, and Oörni K. Decrease in pH strongly enhances binding of native, proteolyzed, lipolyzed, and oxidized low density lipoprotein particles to human aortic proteoglycans. *J Biol Chem.* 2005;280(45):37449-54.
11. Steffen HLM, Anderson JLC, Poot ML, Lei Y, Connelly MA, Bakker SJL, et al. Proteoglycan binding as proatherogenic function metric of apoB-containing lipoproteins and chronic kidney graft failure. *J Lipid Res.* 2021;62:100083.
12. Hurt-Camejo E, Camejo G, Rosengren B, Lopez F, Wiklund O, and Bondjers G. Differential uptake of proteoglycan-selected subfractions of low density lipoprotein by human macrophages. *Journal of lipid research.* 1990;31(8):1387-98.
13. Ruuth M, Nguyen SD, Vihervaara T, Hilvo M, Laajala TD, Kondadi PK, et al. Susceptibility of low-density lipoprotein particles to aggregate depends on particle lipidome, is modifiable, and associates with future cardiovascular deaths. *Eur Heart J.* 2018;39(27):2562-73.
14. Ruuth M, Janssen LGM, Äikäs L, Tigistu-Sahle F, Nahon KJ, Ritvos O, et al. LDL aggregation susceptibility is higher in healthy South Asian compared with white Caucasian men. *J Clin Lipidol.* 2019;13(6):910-9.e2.
15. Äikäs L, Kovanen PT, Lorey M, Laaksonen R, Holopainen M, Ruhanen H, et al. Remodelling of plasma lipoproteins by icosapent ethyl -supplementation and its impact on cardiovascular disease risk markers in normolipidemic individuals. *medRxiv.* 2024:2024.11.27.24318042.
16. House AH, Debes PV, Holopainen M, Käkelä R, Donner I, Frapin M, et al. Seasonal and genetic effects on lipid profiles of juvenile Atlantic salmon. *Biochim Biophys Acta Mol Cell Biol Lipids.* 2025;1870(1):159565.

17. Breitkopf SB, Ricoult SJH, Yuan M, Xu Y, Peake DA, Manning BD, et al. A relative quantitative positive/negative ion switching method for untargeted lipidomics via high resolution LC-MS/MS from any biological source. *Metabolomics.* 2017;13(3).
18. Haimi P, Uphoff A, Hermansson M, and Somerharju P. Software tools for analysis of mass spectrometric lipidome data. *Anal Chem.* 2006;78(24):8324-31.
